# GPR65 Functions as a Key Factor of Bone Aging and a Novel Therapeutic Target for Osteoporosis

**DOI:** 10.1111/acel.70212

**Published:** 2025-09-02

**Authors:** Kun Zhang, Yehua Li, Yi Ren, Yifan He, Jiajun Wang, Xiaoxiu Li, Kefei Guo, Yi Yang, Zhemin Shi, Lina Zheng, Wei Hong

**Affiliations:** ^1^ Department of Histology and Developmental Biology, School of Basic Medical Sciences Tianjin Medical University Tianjin China

**Keywords:** GPCR, GPR65, gαq, osteoclast differentiation, osteoporosis

## Abstract

Osteoporosis (OP) is a metabolic bone disease, characterized by loss of bone mass and destruction of bone microstructure, which has a high incidence of disability. Identification of the key factors of pathogenesis is essential for diagnosis and therapy. In this study, we have identified the proton‐sensing receptor GPR65, which is specifically expressed in osteoclasts and is significantly down‐expressed in osteoclast differentiation, aging, ovariectomy (OVX)‐, and tail suspension (TS)‐induced osteoporotic bone tissue. In vivo experiments confirmed that knockout of GPR65 exacerbates bone loss and OP induced by TS, OVX, and aging. In vitro experiments demonstrated that silencing GPR65 or application of either endogenous or exogenous antagonist of GPR65 promotes osteoclast differentiation, whereas overexpression of GPR65 or application of either endogenous or exogenous agonist inhibits osteoclast differentiation, and knockout of Gpr65 mitigates this effect. Mechanistic studies revealed that GPR65 inhibits osteoclast differentiation by binding to Gαq, activating GSK3β, and suppressing its phosphorylation, thereby inhibiting the nuclear translocation of NFATc1 that mediates osteoclast differentiation. Furthermore, application of GPR65 agonist alleviated OVX‐induced OP in vivo, indicating GPR65 as a novel therapeutic target for bone aging and OP.

## Graphical abstract

This study demonstrates that osteoclast‐enriched GPR65 inhibits osteoclast differentiation and bone loss through the Gαq‐GSK3β‐NFATc1 signaling pathway. The application of the GPR65 agonist is found to alleviate bone loss and osteoporosis, providing a potential specific therapeutic target for bone aging and osteoporosis.

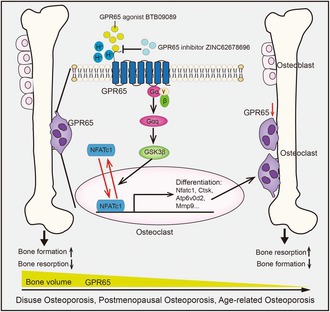

## 1 Introduction

Osteoporosis (OP) refers to a disease caused by the imbalance between osteoclast‐mediated bone resorption and osteoblast‐mediated bone formation, characterized by reduced bone mass and deteriorated microarchitecture of bone tissue, leading to increased bone fragility, with high rates of morbidity and mortality (Bai et al. 2025; Chan et al. 2024; Vandenbroucke et al. 2017). According to statistics, the annual cost of OP treatment in the United States reaches up to $17 billion, while in China, over 100 million individuals suffer from OP, with nearly 100% of women aged over 70 affected by the disease (Imai et al. 2017; Vidal et al. 2019). Fractures in elderly patients with OP impose a severe economic burden and social problems on both the patients themselves and society. Despite the development of therapeutic drugs for OP, clinical application remains limited (Vidal et al. 2019). Therefore, an in‐depth exploration of the molecular mechanisms underlying OP can provide theoretical and experimental evidence for the development of precision treatment drugs for OP.

G‐protein‐coupled receptors (GPCRs), also known as seven‐transmembrane domain receptors, are closely associated with diabetes, obesity, cardiovascular diseases, and cancer, making them highly attractive therapeutic targets for drug development worldwide over the past few decades (Liu et al. 2024). Indeed, approximately 34% of all drugs approved by the Food and Drug Administration (FDA) target GPCRs. GPR65, also known as T cell death‐associated gene 8 (TDAG8), is a proton‐sensitive GPCR that was initially found to be highly expressed in the immune system and to affect thymocyte apoptosis (Radu et al. 2005; Wang et al. 2004). Previous studies have shown that GPR65, upon activation by extracellular acid, triggers the accumulation of intracellular cAMP (Ihara et al. 2010). Im et al. reported that psychosine (PSY) can activate GPR65; however, they did not provide definitive evidence of the specific binding between PSY and GPR65, and subsequent studies indicated that GPR65 is not involved in PSY‐induced multinucleated cell formation (Im et al. 2001; Radu et al. 2006). In an effort to identify more specific ligands for GPR65, Oda et al. identified a compound named “BTB09089” as a specific agonist of GPR65 through library screening (Onozawa et al. 2012). Roth et al. further confirmed “BTB09089” as a potent agonist for GPR65 using a yeast‐based screening method and identified a novel compound named “ZINC62678696” as a potent and specific inhibitor of GPR65 (Huang et al. 2015).

Significant advances have been made in research on GPR65 across multiple fields, including colitis, cancer, and liver fibrosis (Dai et al. 2020; Ihara et al. 2010; Li et al. 2023; Li et al. 2013; Morales Rodriguez et al. 2023; Neale et al. 2024; Zhang et al. 2023). GPR65 serves as a regulatory factor for pain caused by inflammation and cancer, and its antagonists are effective therapies for inflammatory and neuropathic pain (Dai et al. 2020). Studies have revealed that GPR65 and its inflammation‐related Ile231Leu coding variant exhibit cell‐type‐specific roles in inflammation. GPR65 Ile231Leu knock‐in mice display high susceptibility to T‐cell‐driven colitis induced by bacterial infection (Li et al. 2023; Neale et al. 2024). In the context of tumor diseases, GPR65 exhibits both tumor‐promoting and tumor‐suppressing effects (Ihara et al. 2010). It has been found that overexpression of GPR65 promotes the proliferation of non‐small‐cell lung cancer cells and enhances their resistance to an acidic milieu, while knockdown of GPR65 reverses these phenotypes (Ihara et al. 2010). On the other hand, GPR65 exerts tumor‐suppressive properties in malignant hematological diseases by inhibiting the expression of the oncogene c‐Myc (Li et al. 2013). Additionally, our previous research has discovered that knockout of Gpr65 significantly alleviates liver inflammation, injury, and fibrosis (Zhang et al. 2023). In the field of skeletal diseases, although GPR65 has been shown to potentially regulate bone resorption (Chen et al. 2021; Hikiji et al. 2014), its expression profile, function, and the underlying mechanism of action in the process of OP and osteoclast differentiation remain unclear.

The present study has identified that GPR65 is specifically expressed in osteoclasts within bone tissue and is significantly downregulated in the femurs of osteoporotic mice induced by aging, ovariectomy (OVX), and tail suspension (TS). Multiple in vivo and in vitro models have confirmed that GPR65 inhibits osteoclast differentiation through the Gαq‐GSK3β‐NFATc1 signaling pathway, and the application of the GPR65 agonist was found to alleviate OP induced by OVX, providing a potential specific target for the treatment of OP.

## 2 Results

### 2.1 GPR65 Is Enriched in Osteoclasts but Is Significantly Downregulated in Osteoclast Differentiation and Osteoporotic Bone Tissue

**FIGURE 1:**
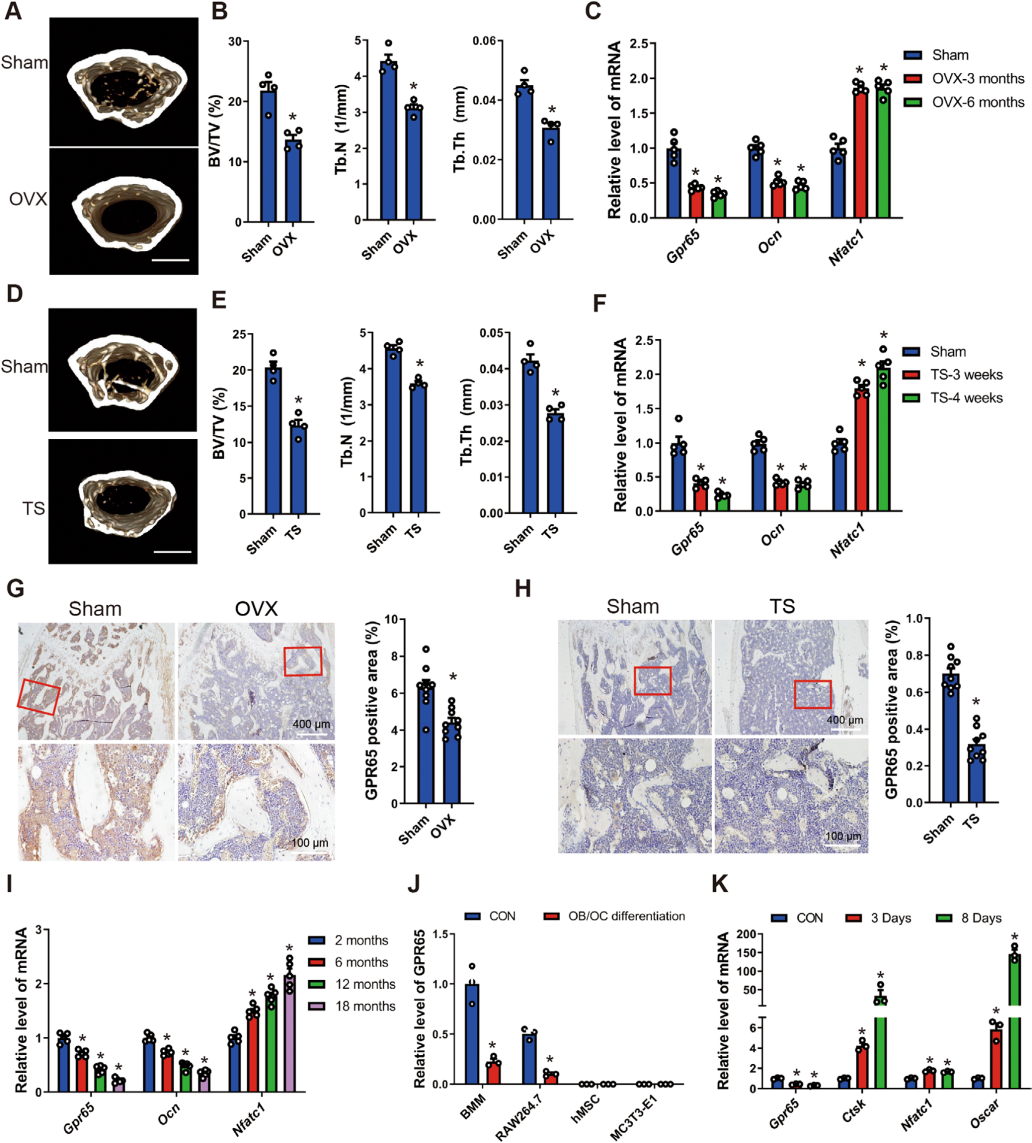
GPR65 is enriched in osteoclasts but is significantly downregulated in osteoclasts differentiation and osteoporotic bone tissue. (A, B) Micro‐CT analysis of distal femurs from Sham and ovariectomy (OVX) mice for bone volume per tissue volume (BV/TV), trabecular number (Tb.N), and trabecular thickness (Tb.Th); scale bar, 500 μm (*n* = 4). (C) qRT‐PCR analysis of *Gpr65*, *Ocn* and *Nfatc1* in osteoporotic bone tissues induced by OVX for 3 and 6 months (*n* = 5). (D, E) Micro‐CT analysis of distal femurs from Control and tail suspension (TS) mice for BV/TV, Tb.N and Tb.Th, scale bar, 500 μm (*n* = 4). (F) qRT‐PCR analysis of *Gpr65*, *Ocn* and *Nfatc1* in osteoporotic bone tissues induced by TS for 3 and 4 weeks (*n* = 5). (G, H) Representative IHC staining illustrating GPR65 expression in the mice femurs with or without OVX and TS treatment; scale bar, 100 μm for 40× and 400 μm for 10×. Quantifications are shown in the right panel (*n* = 9 images taken in total; three images from each of three biological replicates). (I) qRT‐PCR analysis of *Gpr65*, *Ocn* and *Nfatc1* in bone tissue from WT mice at indicated ages (*n* = 5). (J) qRT‐PCR analysis of *Gpr65* in osteogenic and osteoclastogenic induction (*n* = 3). (K) qRT‐PCR analysis of *Gpr65*, *Ctsk*, *Nfatc1*, and *Oscar* in primary mouse bone marrow macrophages (BMM) cultured in osteoclast differentiation medium for 3 or 8 days (*n* = 3). *p < 0.05 for versus Sham, 2 months or CON.

To explore whether GPCRs play crucial roles in osteoclast differentiation and the progression of OP, we screened three osteoclast differentiation‐related datasets, GSE72047, GSE74847, and GSE225974, from the GEO database. By intersecting the differentially expressed genes in osteoclast differentiation with the GPCR dataset, we have identified Gpr65 as a significantly abnormally expressed receptor in this process (Figure S1A–G). Subsequently, we have detected the expression of Gpr65 in the femoral tissues of three osteoporotic mouse models induced by TS, OVX, and aging, respectively, and found GPR65 was significantly down‐expressed in the osteoporotic bone tissue (Figure 1A–I). Next, we examined the expression of Gpr65 in osteogenic and osteoclastogenic induction, and the results revealed GPR65 level was minimal in pre‐osteoblasts and kept unchanged in the osteogenic induction of hMSCs and MC3T3‐E1 cells (Figure 1J). However, Gpr65 was enriched in BMMs and osteoclasts derived from BMM and RAW264.7 cells, and its expression decreased gradually over the duration of osteoclastogenic induction (Figure 1K and Figure S1H). Taken together, GPR65 is enriched in osteoclasts but is significantly downregulated in osteoclast differentiation and OP.

### 2.2 Knockout of Gpr65 Exhibits Age‐Related Bone Loss and OP

**FIGURE 2:**
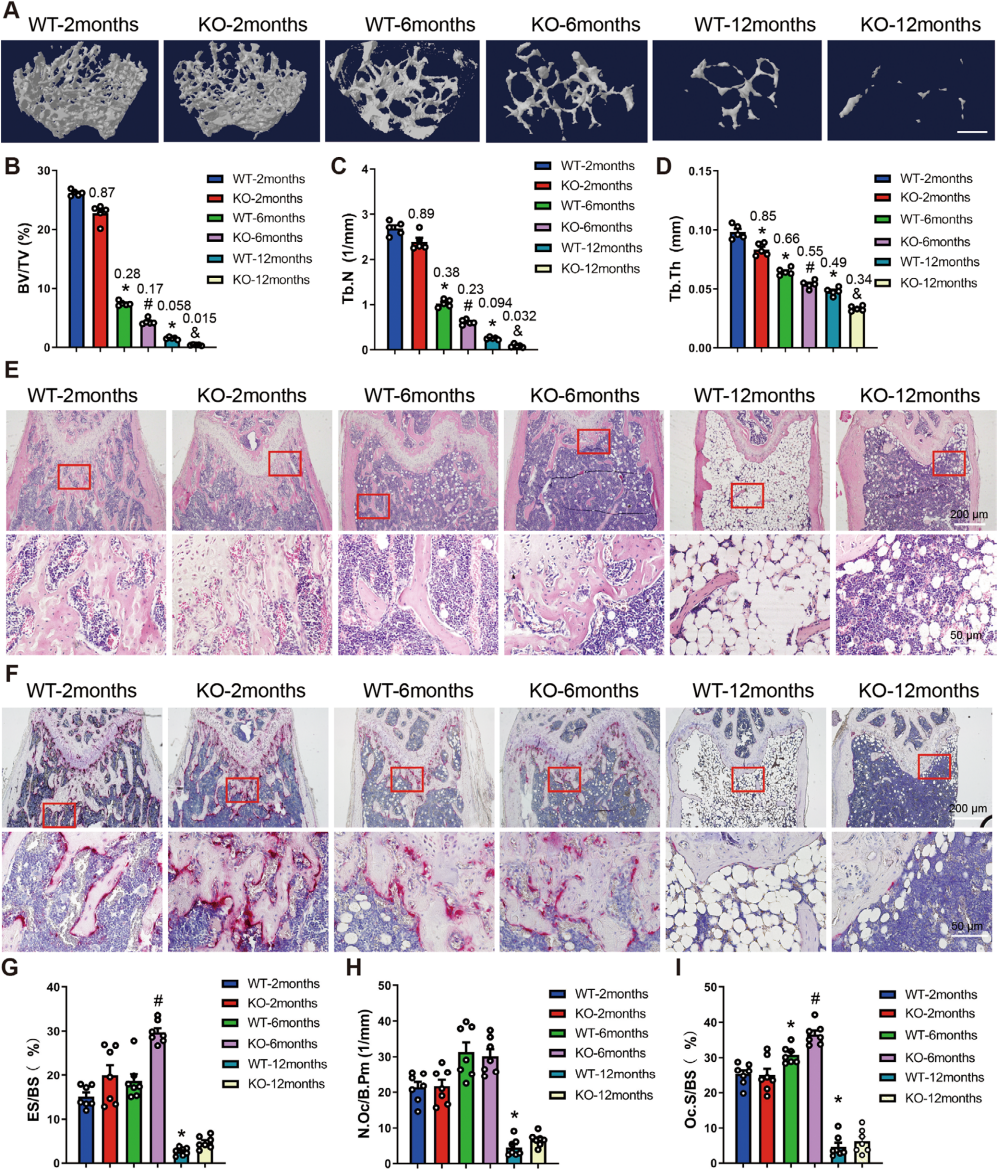
Deletion of Gpr65 results in age‐related bone loss and OP. (A–D) Micro‐CT analysis of distal femurs from 2‐, 6‐ and 12‐month‐old female WT and GPR65 KO mice for BV/TV, Tb.N and Tb.Th, scale bar, 500 μm; values in the figure represent ratios relative to WT‐2 months (*n* = 5). (E) Representative HE staining images of femurs from 2‐, 6‐ and 12‐month‐old female WT and GPR65 KO mice; scale bar, 50 μm for 20× and 200 μm for 4×. (F–I) Representative TRAP staining images (F) and parameters (G–I) of femur osteoclasts from 2‐, 6‐ and 12‐month‐old female WT and GPR65 KO mice. ES/BS, eroded surface per bone surface; N.Oc/B.Pm, number of osteoblasts per bone perimeter; Oc.S/BS, osteoclast surface per bone surface; scale bar, 50 μm for 20× and 200 μm for 4×. *p < 0.05 for versus WT‐2 months. ^#^ *p* < 0.05 for versus WT‐6 months. ^&^ *p* < 0.05 for versus WT‐12 months.

As the elderly population is the primary susceptible group for OP, we generated GPR65 KO mice, which were confirmed by qRT‐PCR and DNA sequencing, and used these mice to establish a natural aging‐induced OP model to investigate the role of GPR65 in age‐related OP. Micro‐CT analysis revealed that older WT mice exhibited a gradual decrease in trabecular bone volume (BV/TV), trabecular number (Tb.N) and trabecular thickness (Tb.Th), accompanied by a gradual increase in trabecular bone pattern factor (Tb. Pf) and structure model index (SMI). When compared to the age‐matched WT groups, the GPR65 KO‐6 month and GPR65 KO‐12 month groups had further reductions in BV/TV, Tb.N, and Tb.Th, and an increase in Tb. Pf (Figure 2A–D; Figure S2A,B). However, the GPR65 KO‐2 month group only showed a further reduction in trabecular thickness (Figure 2A–D; Figure S2A,B). ELISA results indicated that compared to the age‐matched WT groups, the GPR65 KO groups exhibited significantly increased levels of the bone‐resorption (osteoclast) markers CTX and TRAP5b in serum, while no significant changes were observed in bone‐formation (osteoblast) markers PINP and OCN (Figure S2C–F). Furthermore, bone‐morphometric analysis of HE and TRAP staining revealed that GPR65 KO mice showed reduced trabecular number and thickness, accompanied by increased osteoclast surface and eroded surface at 6 months of age, compared with the age‐matched WT groups (Figure 2E–I). In addition, Micro‐CT analysis of lumbar vertebral trabecular bone and femoral cortical bone from 6‐month‐old female WT and GPR65 KO mice further demonstrated that GPR65 KO mice exhibited significant reductions in lumbar vertebral trabecular BV/TV and Tb.N, as well as femoral cortical bone mineral density (BMD) and mean total cross‐sectional bone area (B.Ar) (Figure S2G–N). In summary, these results indicate that GPR65 knockout exhibits age‐related bone loss and OP.

### 2.3 Knockout of Gpr65 Exacerbates TS‐ and OVX‐Induced OP

**FIGURE 3:**
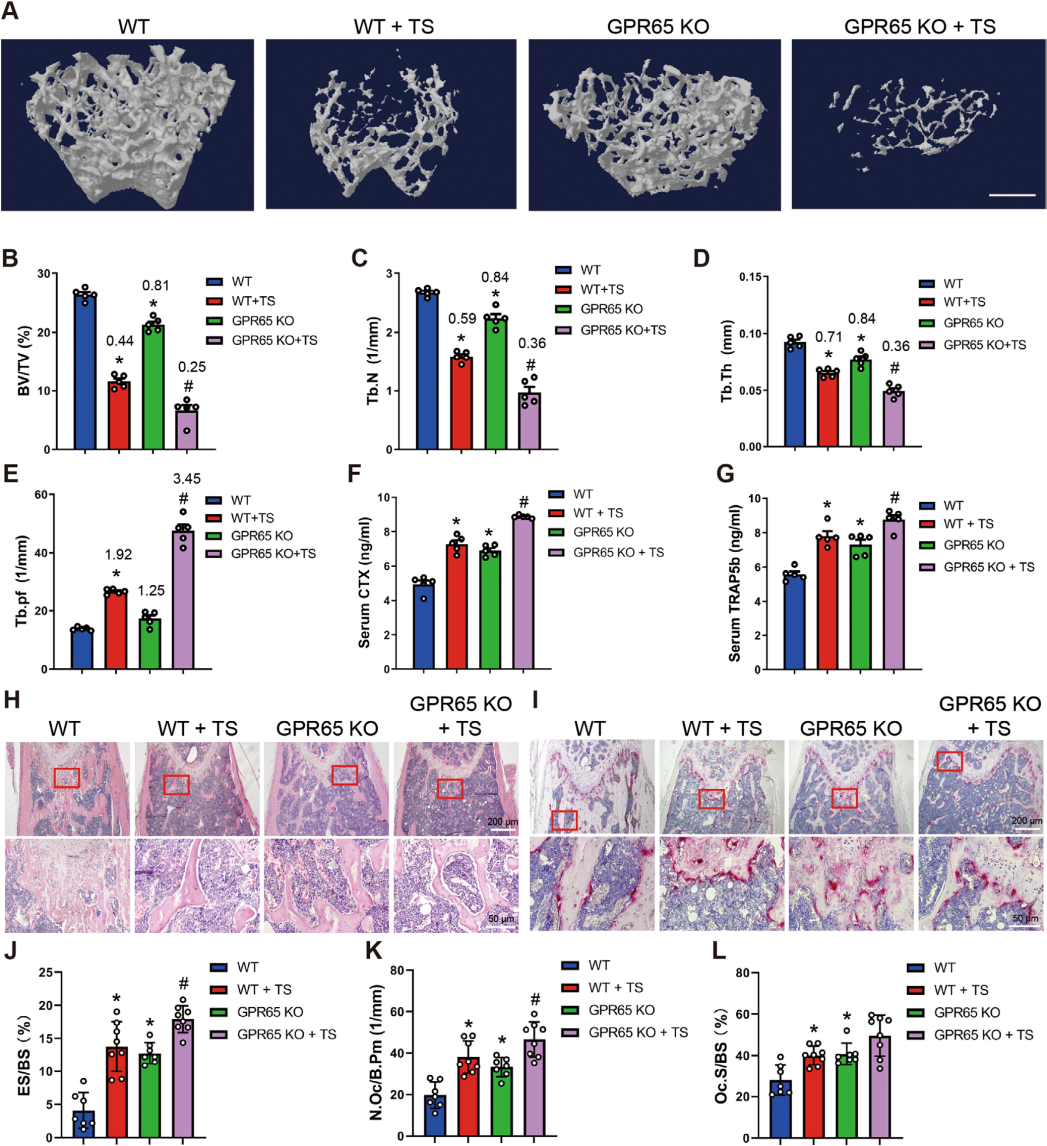
Knockout of Gpr65 exacerbates TS‐induced OP. (A–E) Micro‐CT analysis of distal femurs from WT and GPR65 KO male mice at 8 weeks with or without TS treatment for BV/TV, Tb.N, Tb.Th and Tb. Pf, scale bar, 500 μm; values in the figure represent ratios relative to WT (*n* = 5). (F, G) Serum CTX and TRAP5b levels in WT and GPR65 KO mice with or without TS treatment. (H, I) Representative HE staining images and TRAP staining images of femurs from WT and GPR65 KO mice with or without TS treatment; scale bar, 50 μm for 20× and 200 μm for 4×. (J–L) Bone‐morphometric analysis of femurs from WT and GPR65 KO mice with or without TS treatment for ES/BS, N.Oc/B.Pm and Oc.S/BS. *p < 0.05 for versus WT. ^#^ *p* < 0.05 for versus WT + TS.

To further investigate the function of Gpr65 in disuse osteoporosis in vivo, we established a TS‐induced OP model using GPR65 KO and WT mice. Micro‐CT analysis revealed that TS led to decreased BV/TV, Tb.N, and Tb.Th but increased Tb. Pf and SMI compared to the control group. Knockout of GPR65 further decreased BV/TV, Tb.N, and Tb.Th, but increased Tb. Pf, indicating exacerbated OP (Figure 3A–E and Figure S3A). Furthermore, the serum bone resorption markers CTX and TRAP5b were significantly higher in TS‐treated GPR65 KO mice than in TS‐treated WT mice (Figure 3F,G), whereas no significant difference was observed in the serum bone formation markers PINP and OCN (Figure S3B,C). In addition, bone morphometric analysis of HE and TRAP staining further revealed that TS‐treated GPR65 KO mice exhibited significantly increased osteoclast number and eroded surface, but not osteoclast surface, compared to TS‐treated WT mice (Figure 3H–L).

**FIGURE 4:**
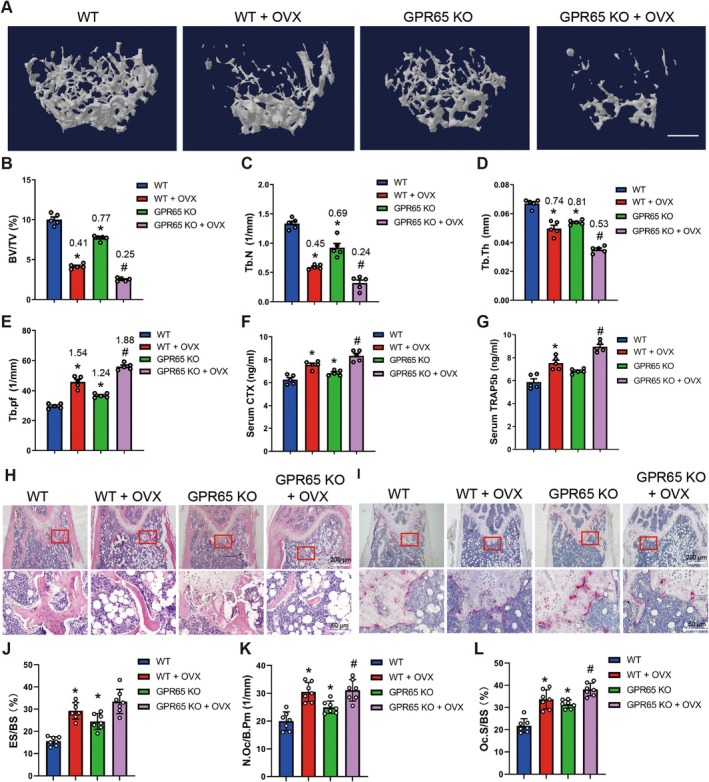
Knockout of Gpr65 exacerbates OVX‐induced OP. (A–E) Micro‐CT analysis of distal femurs from WT and GPR65 KO female mice at 12 weeks with or without OVX treatment for BV/TV, Tb.N, Tb.Th and Tb. Pf, scale bar, 500 μm; values in the figure represent ratios relative to WT (*n* = 5). (F, G) Serum CTX and TRAP5b levels in WT and GPR65 KO mice with or without OVX treatment. (H, I) Representative HE staining images and TRAP staining images of femurs from WT and GPR65 KO mice with or without OVX treatment; scale bar, 50 μm for 20× and 200 μm for 4×. (J–L) Bone‐morphometric analysis of femurs from WT and GPR65 KO mice with or without OVX treatment for ES/BS, N.Oc/B.Pm and Oc.S/BS. *p < 0.05 for versus WT. ^#^ *p* < 0.05 for versus WT + OVX.

Given that postmenopausal osteoporosis is the most common type of OP, we used GPR65 KO mice to establish an OVX‐induced OP mouse model to investigate whether GPR65 is also involved in estrogen deficiency‐induced OP. Micro‐CT analysis revealed that OVX resulted in decreased BV/TV, Tb.N, and Tb.Th, but increased Tb. Pf and SMI, compared to the control group. Moreover, knockout of GPR65 further decreased BV/TV, Tb.N, and Tb.Th, but increased Tb. Pf (Figure 4A–E and Figure S4A). Also, OVX‐treated GPR65 KO mice exhibited significantly increased serum CTX and TRAP5b, but not serum bone formation markers, compared to OVX‐treated WT mice (Figure 4F,G; Figure S4B,C). In addition, bone‐morphometric analysis further revealed loss of Gpr65 significantly promoted the increased osteoclast number and osteoclast surface induced by OVX (Figure 4H–L). In conclusion, our data indicate that knockout of Gpr65 exacerbates TS‐ and OVX‐induced OP.

### 2.4 GPR65 Inhibits Osteoclast Differentiation

**FIGURE 5:**
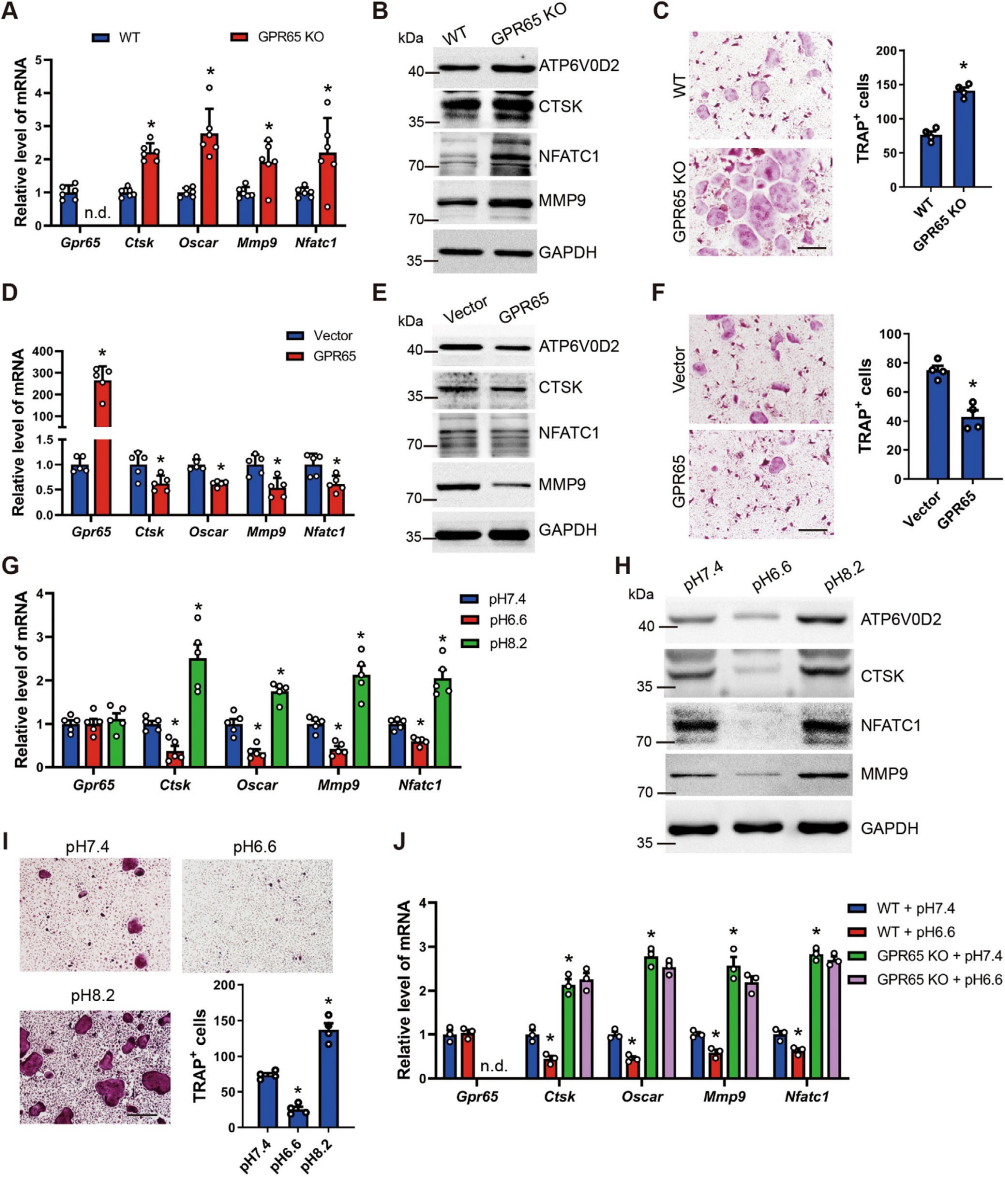
GPR65 inhibits osteoclasts' differentiation. (A) qRT‐PCR analysis of *Gpr65*, *Ctsk*, *Oscar*, *Mmp9* and *Nfatc1* in WT and GPR65 KO BMM cultured in osteoclast differentiation medium for 8 days (*n* = 6). (B) Western blot of ATP6V0D2, CTSK, NFATc1 and MMP9 in WT and GPR65 KO BMM cultured in osteoclast differentiation medium for 8 days. GAPDH was used as an internal control. (C) Representative TRAP staining images of WT and GPR65 KO BMM cultured in osteoclast differentiation medium for 8 days; scale bar, 200 μm. Quantifications are shown in the right panel (*n* = 4). (D–F) BMM were transfected with pcDNA3.1‐GPR65 or pcDNA3.1 and simultaneously cultured in osteoclast differentiation medium for 8 days. qRT‐PCR analysis of the expression of *Gpr65*, *Ctsk*, *Oscar*, *Mmp9*, and *Nfatc1* (D) (*n* = 5). The protein level of ATP6V0D2, CTSK, NFATc1, and MMP9 was determined by western blot (E). GAPDH was used as an internal control. Representative TRAP staining images of osteoclasts (F) (*n* = 4); scale bar, 200 μm. Quantifications are shown in the right panel. (G–I) BMM were incubated in the physiological pH 7.4, the acidic pH 6.6, or the alkaline pH 8.2 and simultaneously cultured in osteoclast differentiation medium for 8 days. qRT‐PCR analysis of the expression of *Gpr65*, *Ctsk*, *Oscar*, *Mmp9* and *Nfatc1* (G) (*n* = 4). The protein level of ATP6V0D2, CTSK, NFATc1, and MMP9 was determined by western blot (H). GAPDH was used as an internal control. Representative TRAP staining images of osteoclasts (I) (*n* = 4); scale bar, 200 μm. Quantifications are shown in the right panel. (J) WT and GPR65 KO BMM were incubated in pH 7.4 and pH 6.6 and simultaneously cultured in osteoclast differentiation medium for 8 days. qRT‐PCR analysis of the expression of *Gpr65*, *Ctsk*, *Oscar*, *Mmp9*, and *Nfatc1* (*n* = 3). *p < 0.05 for versus WT or Vector or pH 7.4 or WT + pH 7.4.

GPR65 is enriched in osteoclasts and is involved in OP, leading us to probe whether GPR65 plays a role in osteoclast differentiation. Therefore, we isolated primary BMM from WT and GPR65 KO mice, respectively, to induce the cells with M‐CSF and RANKL for 8 days. Knockout of GPR65 promoted the expression of osteoclast‐related genes including CTSK, OSCAR, MMP9, NFATc1, and ATP6V0D2, as shown by the results of qRT‐PCR and Western blot (Figure 5A,B). Moreover, TRAP staining confirmed that the number and area of TRAP‐positive cells were significantly increased when GPR65 was deleted (Figure 5C). Subsequently, overexpression of GPR65 in primary BMM from WT mice with pcDNA3.1‐GPR65 inhibited osteoclast‐related gene expression (Figure 5D,E). TRAP staining further demonstrated that GPR65 overexpression reduced the number and area of TRAP‐positive cells (Figure 5F). These findings were further confirmed in RAW264.7 cells (Figure S5A–F). On the other hand, despite the low abundance of GPR65 in pre‐osteoblasts, we overexpressed GPR65 in these cells and induced osteoblast differentiation. qPCR and ALP staining results indicated that overexpression of GPR65 did not affect osteoblast differentiation (Figure S6A,B). In conclusion, these results indicate that GPR65, which is enriched in osteoclasts, inhibits osteoclast differentiation.

### 2.5 Extracellular Acidification Inhibits Osteoclast Differentiation via GPR65

As GPR65 has been accepted as a proton‐sensing GPCR and the extracellular acidic environment plays a crucial role in osteoclast activity (Komarova et al. 2005; Pereverzev et al. 2008), we therefore investigated the effects of extracellular acidification on osteoclast differentiation and whether GPR65 gets involved. We isolated mouse BMM culturing in media with various pH, including the physiological pH 7.4, the alkaline pH 8.2, and the acidic pH 6.6, to induce osteoclast differentiation. The results showed that the extracellular acidic environment inhibits, while the alkaline environment promotes the gene expression of osteoclast differentiation (Figure 5G,H). TRAP staining further confirmed that extracellular acidification reduced the number and area of TRAP‐positive osteoclasts, whereas extracellular alkalinization showed an opposite effect (Figure 5I). We further used BMM from WT and GPR65 KO mice for the induction of osteoclast differentiation under conditions of pH 7.2 and pH 6.6, respectively. The results showed that GPR65 knockout abolished the inhibitory effect of extracellular acidification on osteoclast differentiation (Figure 5J; Figure S7A,B). These findings were also confirmed in RAW264.7 cells (Figure S8A–F). In summary, the acidic extracellular environment inhibits osteoclast differentiation through GPR65.

### 2.6 Effect of GPR65 Exogenous Agonist and Inhibitor on Osteoclast Differentiation

It has been reported that BTB09089 is an allosteric agonist and ZINC62678696 functions as an inhibitor of GPR65 (Huang et al. 2015). To further confirm the role of GPR65 in osteoclast differentiation, we treated BMM with BTB09089 or ZINC62678696 when the cells were induced for osteoclast differentiation, and the results revealed that BTB09089 significantly inhibited, while ZINC62678696 promoted the expression of genes related to osteoclast differentiation, as well as the number and area of TRAP‐positive osteoclasts (Figure S9A–C). Further experiments also confirmed that the knockout of Gpr65 abolished the inhibitory effect of BTB09089 on osteoclast differentiation (Figure S9D–F). These findings were further confirmed in RAW264.7 cells (Figure S10A–F). Taken together, these data indicate that the GPR65 exogenous agonist inhibits, while the GPR65 inhibitor promotes osteoclast differentiation.

### 2.7 Gαq Mediates GPR65 Function

**FIGURE 6:**
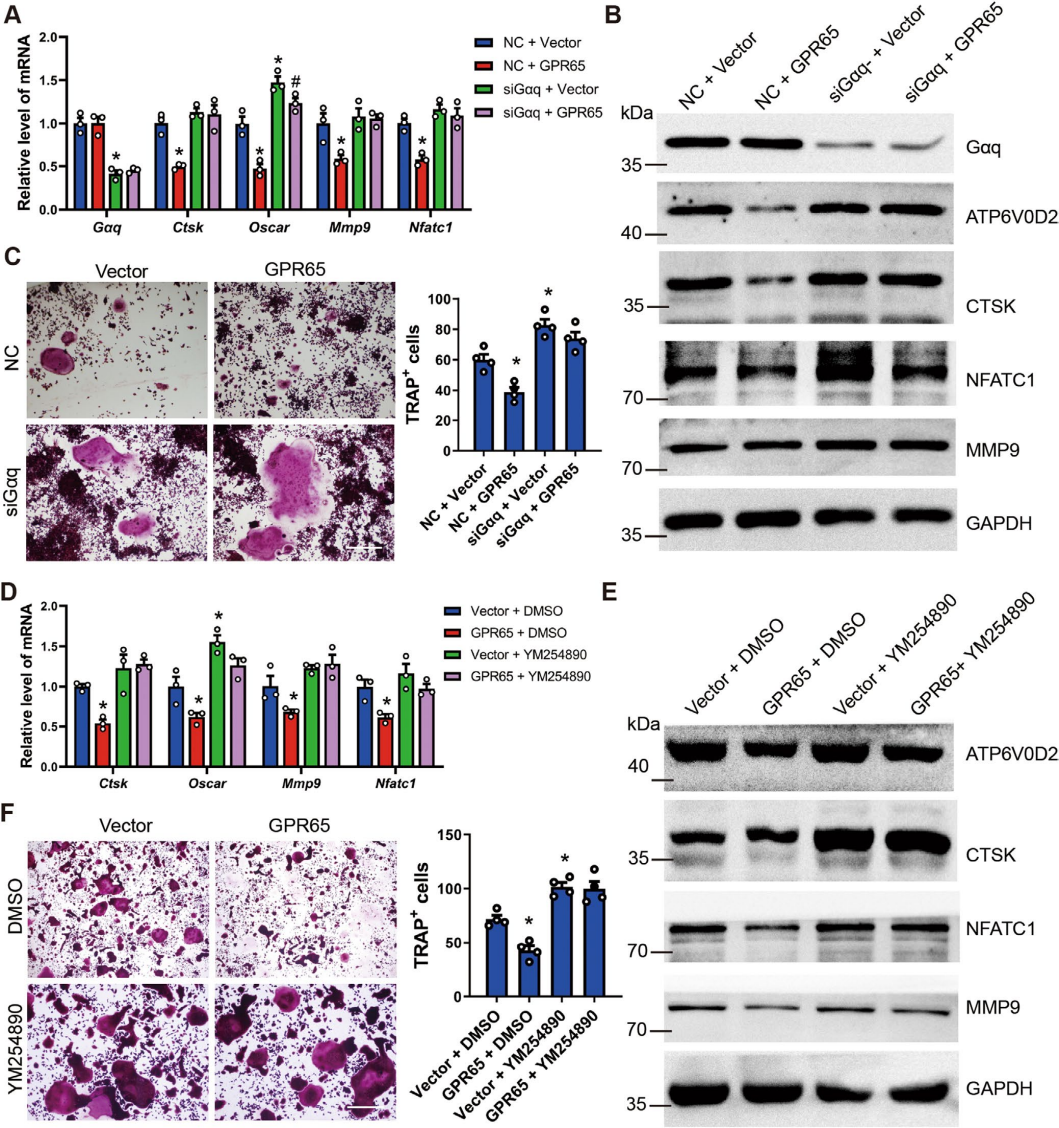
GPR65 inhibits osteoclast differentiation through Gαq. (A–C) Control and GPR65‐overexpressed RAW264.7 cells were transfected with Gαq‐siRNA or negative control, and simultaneously cultured in osteoclast differentiation medium (3 days for qRT‐PCR and Western blot; 5 days for TRAP staining). qRT‐PCR analysis of the expression of *Gαq*, *Ctsk*, *Oscar*, *Mmp9*, and *Nfatc1* (A) (*n* = 3). The protein level of Gαq, ATP6V0D2, CTSK, NFATc1, and MMP9 was determined by western blot (B). GAPDH was used as an internal control. Representative TRAP staining images of osteoclasts (C); scale bar, 200 μm. Quantifications are shown in the right panel (*n* = 4). (D–F) Control and GPR65‐overexpressed RAW264.7 cells were treated with or without 1 μM Gαq inhibitor YM254890, and simultaneously cultured in osteoclast differentiation medium (3 days for qRT‐PCR and Western blot; 5 days for TRAP staining). qRT‐PCR analysis of the expression of *Ctsk*, *Oscar*, *Mmp9*, and *Nfatc1* (D) (*n* = 3). The protein level of ATP6V0D2, CTSK, NFATc1, and MMP9 was determined by western blot (E); GAPDH was used as an internal control. Representative TRAP staining images of osteoclasts (F); scale bar, 200 μm. Quantifications are shown in the right panel (*n* = 4). *p < 0.05 for versus NC + Vector or Vector + DMSO.

GPCRs typically regulate downstream pathways by binding to G proteins, and previous research has found that GPR65 binds to Gαq and Gαs (Kottyan et al. 2009; Zhang et al. 2023). Among them, Gαq is known to inhibit osteoclast differentiation (Chakraborty et al. 2022; Luo et al. 2016), leading us to hypothesize that GPR65 binds to Gαq to trigger downstream pathways, thereby inhibiting osteoclast differentiation. During osteoclast differentiation in RAW264.7 cells, we overexpressed GPR65 while simultaneously silencing Gαq. The results showed that overexpression of GPR65 reduced the expression of osteoclast‐related genes and the number of TRAP‐positive osteoclasts (Figure 6A–C). Conversely, silencing Gαq abolished the repressive effect of GPR65 on osteoclast differentiation (Figure 6A–C). Similarly, the application of Gαq inhibitor YM254890 also released the inhibitory effect of GPR65 overexpression on osteoclast differentiation (Figure 6D–F). We further silenced the expression of Gαq or applied Gαq inhibitor when RAW264.7 cells were induced for differentiation under acidic environments or the application of BTB09089. The results indicated that acidic environments and BTB09089 could reduce the expression of osteoclast‐related genes and the amount of TRAP‐positive osteoclasts (Figures S11A–E and S12A–E). However, silencing Gαq and the application of YM254890 abolished the inhibitory effects of acidification and GPR65 agonist on osteoclast differentiation (Figures S11A–E and S12A–E), suggesting that GPR65 inhibits osteoclast differentiation by binding to Gαq.

### 2.8 GPR65 Inhibits Osteoclast Differentiation by Activating GSK3β, Which in Turn Suppresses the Expression and Nuclear Translocation of NFATc1

**FIGURE 7:**
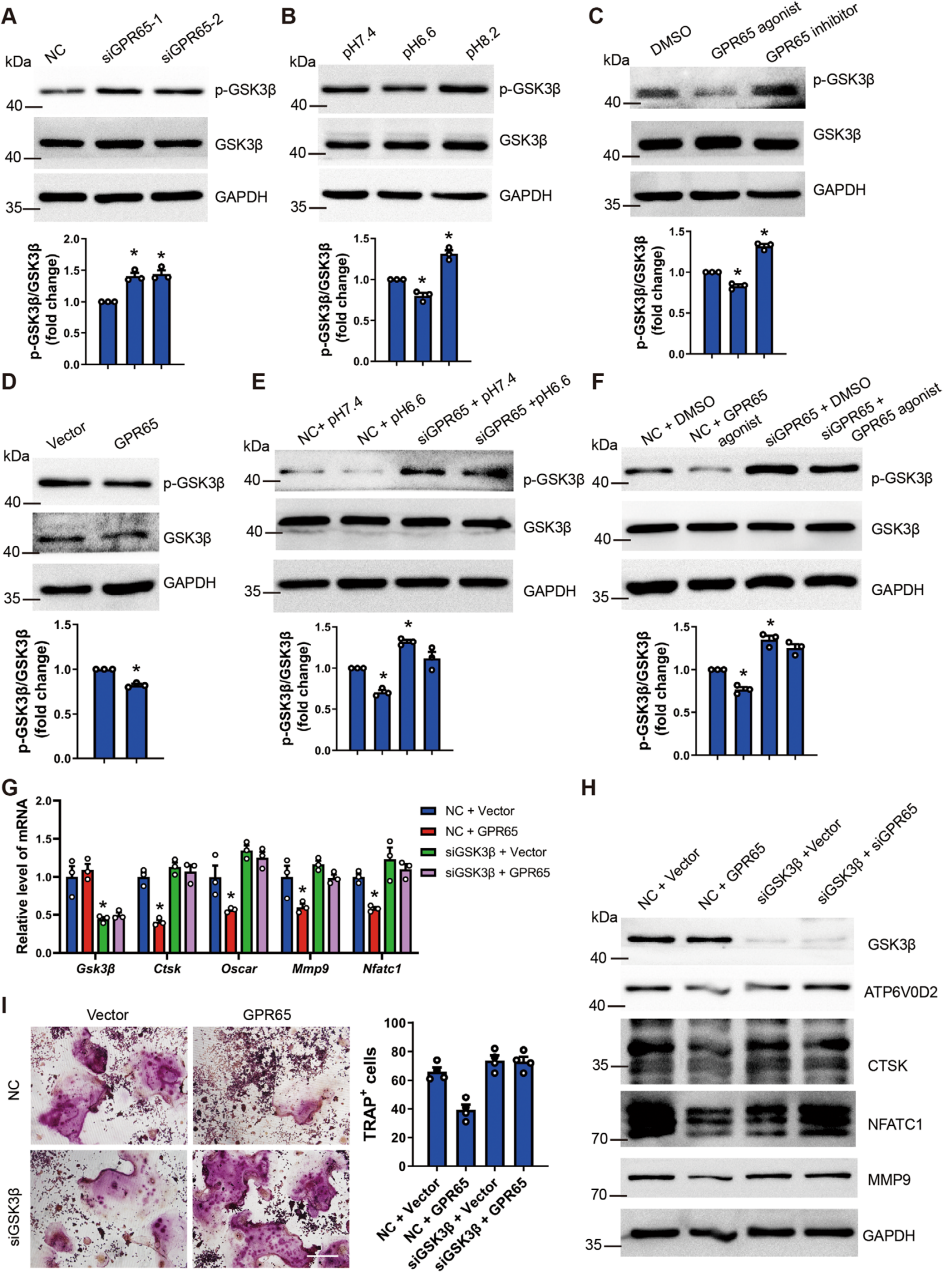
GPR65 inhibits osteoclast differentiation by activating GSK3β. (A–F) Protein level of phos‐GSK3β and total GSK3β in GPR65‐silenced, GPR65‐over‐expressed, various pH‐treated or GPR65 agonist/inhibitor‐treated RAW264.7 cells cultured in osteoclast differentiation medium for 3 days simultaneously was determined by western blot; GAPDH was used as an internal control. Quantifications are shown in the below panel. (G–I) Control and GPR65‐overexpressed RAW264.7 cells were transfected with GSK3β‐siRNA or negative control, and simultaneously cultured in osteoclast differentiation medium (3 days for qRT‐PCR and Western blot; 5 days for TRAP staining). qRT‐PCR analysis of the expression of *Gsk3β*, *Ctsk*, *Oscar*, *Mmp9*, and *Nfatc1* (G) (*n* = 3). The protein level of GSK3β, ATP6V0D2, CTSK, NFATc1, and MMP9 was determined by western blot (H); GAPDH was used as an internal control. Representative TRAP staining images of osteoclasts (I); scale bar, 200 μm. Quantifications are shown in the right panel (*n* = 4). *p < 0.05 for versus NC + Vector.

It has been reported that Gαq can activate GSK3β to inhibit the nuclear translocation of the key transcription factor NFATc1, thereby inhibiting osteoclast differentiation (Luo et al. 2016). Therefore, we first examined the phosphorylation level of GSK3β in BMM and RAW264.7 cells with GPR65 knockout or overexpression, various pH treatments, and treated with GPR65 exogenous agonists and antagonists. Western blot indicated that silencing GPR65, extracellular alkaline environments, and GPR65 exogenous antagonist promoted GSK3β phosphorylation, while GPR65 overexpression, extracellular acidic environments, and GPR65 agonist inhibited GSK3β phosphorylation (Figure 7A–F and Figure S13A–F). Moreover, knockout of GPR65 abolished the inhibitory effect of extracellular acidic environments and GPR65 agonist on GSK3β phosphorylation (Figure 7E,F; Figure S13E,F). Next, we overexpressed GPR65 or treated cells with an acidic environment or GPR65 agonist during the induction of osteoclast differentiation in RAW264.7 cells, while simultaneously silencing GSK3β expression. qRT‐PCR, Western blot, and TRAP staining showed that silencing GSK3β abolished the reduction in osteoclast‐related gene expression and the number of TRAP‐positive osteoclasts caused by GPR65 overexpression, acidic environments, and GPR65 agonist (Figure 7G–I and Figure S14A–E). Finally, we also examined the nuclear–cytoplasmic localization of NFATc1 in BTB09089‐treated Gαq‐silenced or GSK3β‐silenced osteoclasts (RAW264.7 cells induced by RANKL). Confocal results revealed that GPR65 agonist not only induced the translocation of NFATc1 from the nucleus to the cytoplasm but also significantly downregulated the expression of NFATc1 (Figure S15A,B). Silencing Gαq or GSK3β abolished this effect of GPR65 agonist (Figure S15A,B). In summary, GPR65 inhibits osteoclast differentiation by activating GSK3β, which suppresses the expression and nuclear translocation of NFATc1.

### 2.9 Pharmacological GPR65 Activation Alleviates OVX‐Induced OP

**FIGURE 8:**
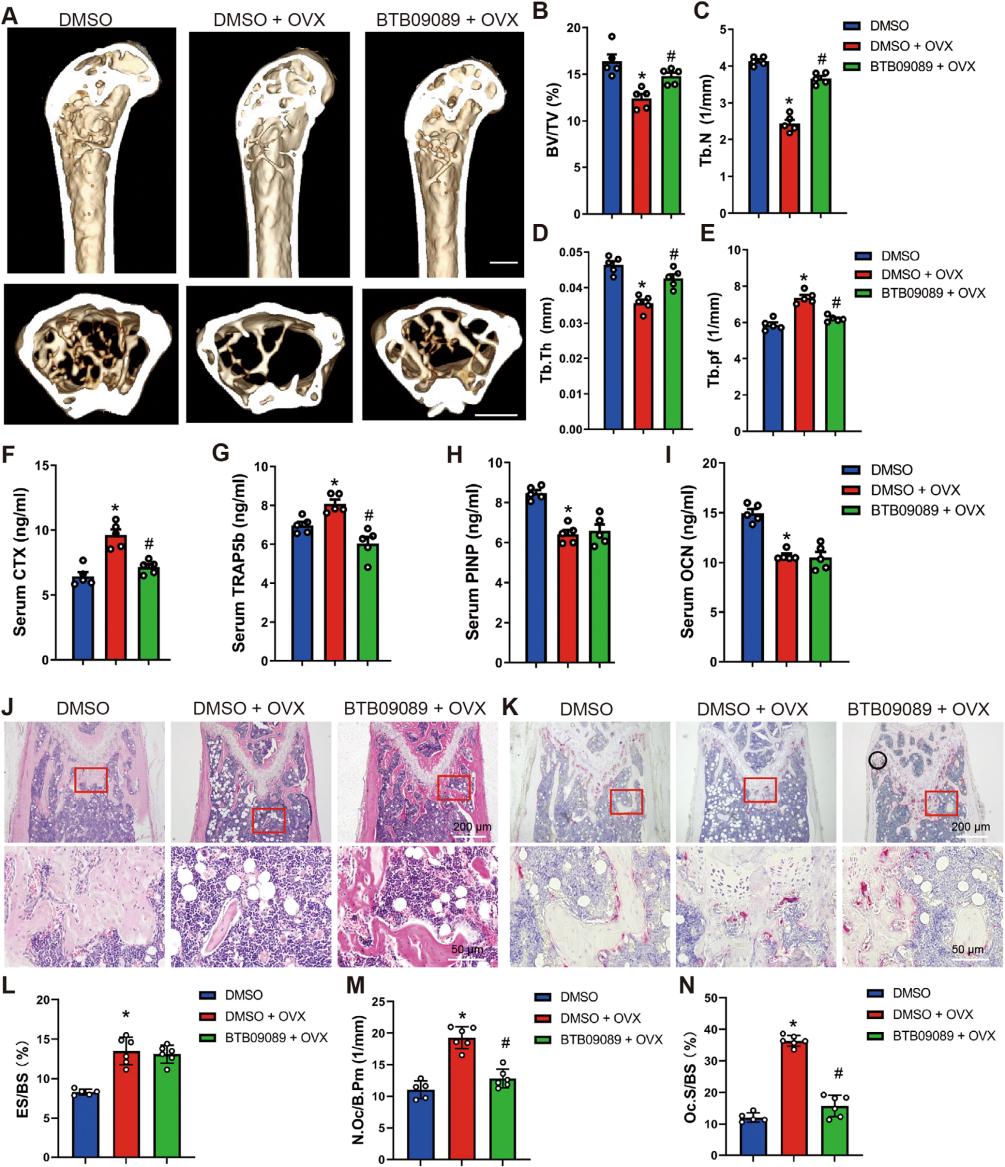
Pharmacological GPR65 activation alleviates OVX‐induced OP. (A–N) Balb/c female mice were separated into three groups randomly: DMSO, DMSO + OVX, BTB09089 + OVX. 10 mg/kg BTB09089 was administered 4 weeks after OVX operation every 2 days for 2 months. Micro‐CT analysis of distal femurs for BV/TV, Tb.N, Tb.Th, and Tb. Pf (A–E) (*n* = 5). Serum CTX, TRAP5b, PINP and OCN levels (F–I) (*n* = 5). Representative HE staining images and TRAP staining images of femurs (J, K); scale bar, 50 μm for 20× and 200 μm for 4×. Bone‐morphometric analysis of femurs for ES/BS, N.Oc/B.Pm and Oc.S/BS (L–N). *p < 0.05 for versus DMSO. ^#^ *p* < 0.05 for versus DMSO + OVX.

To further investigate the clinical application potential of GPR65 in the development of OP, the GPR65 agonist BTB09089 was administered intraperitoneally every 2 days to OVX‐treated or Sham‐treated mice, starting 4 weeks after the OVX operation and continuing for 8 weeks. Our data revealed that intraperitoneal injection of BTB09089 alone does not affect bone metabolism (Figure S16A–I). In contrast, OVX induced a reduction in BV/TV, Tb.N, and Tb.Th, and an increase in Tb. Pf compared to the control group (Figure 8A–E). Notably, the GPR65 agonist BTB09089 significantly inhibited OVX‐induced reduction in relative bone volume fraction, trabecular number, and trabecular thickness (Figure 8A–E). ELISA assays showed that BTB09089‐treated OVX mice had significantly lower serum levels of bone resorption markers CTX and TRAP5b, while no significant changes were observed in bone formation markers PINP and OCN, compared to DMSO‐treated OVX mice (Figure 8F–I). Moreover, bone‐morphometric analysis of HE and TRAP staining further revealed that BTB09089‐treated OVX mice exhibited significantly decreased osteoclast number and osteoclast surface, but not eroded surface, than DMSO‐treated OVX mice (Figure 8J–N). In summary, the application of the GPR65 exogenous ligand improves OVX‐induced OP.

## 3 Discussion

Postmenopausal OP, disuse OP, and age‐related OP represent the primary types of human OP, with distinct mechanisms underlying bone loss in each type (Vandenbroucke et al. 2017; Vidal et al. 2019). For instance, the OVX mouse model, which is a widely used animal model for postmenopausal OP, exhibits increased osteoclast activity due to estrogen deficiency, leading to significantly increased bone resorption. However, it also simultaneously impairs osteoblast function and reduces bone formation. Similarly, the TS model of disuse OP primarily induces bone loss through increased bone resorption. In contrast, age‐related OP in animal models is mainly attributed to reduced bone formation (Komori 2015; Vidal et al. 2019). Our in vivo experiments demonstrated that female GPR65 KO mice begin to exhibit a bone loss phenotype as early as 2 months of age, characterized by reduced trabecular thickness and significantly elevated levels of serum CTX and TRAP5b. At 6 and 12 months of age, the bone loss phenotype in female GPR65 KO mice becomes more pronounced, affecting not only femoral cortical and trabecular bone but also vertebral trabecular bone (Figure 2). Interestingly, male GPR65 KO mice also display significant bone loss at 3 months of age (Figure 3), suggesting that GPR65 deletion‐induced bone loss does not exhibit significant sexual dimorphism. Moreover, in OVX‐ and TS‐induced OP models, we found that GPR65 knockout exacerbates bone loss by more than 20%, suggesting that GPR65 deficiency aggravates OVX‐ and TS‐mediated bone loss. Notably, in both age‐related OP models and OVX/TS‐treated conditions, GPR65 knockout consistently induces bone loss, supporting the conclusion that GPR65 is a critical suppressor of osteoclast differentiation and bone resorption. Downregulation of GPR65 expression significantly enhances osteoclast‐mediated bone resorption, consistent with previous reports that GPR65 inhibits bone resorption (Chen et al. 2021; Hikiji et al. 2014). However, Hikiji et al. (2014) reported that homozygous Gpr65 gene‐trap mice exhibited only a slight enhancement of trabecular bone loss after OVX, but not in non‐OVX mice. Several reasons may account for these discrepancies: (1) Hikiji et al. used mice with a mixed genetic background (BDF1, ICR, and C57BL/6), whereas our study employed a homogeneous C57BL/6 background, which may influence phenotypic outcomes. (2) We employed CRISPR/Cas9 technology to delete most of exon 2 in GPR65, while Hikiji et al. used an insertion mutation that disrupted GPR65 by forcing exon 1 at the 5′ end of the insertion site to splice with the splice acceptor of the Sleeping Beauty transposon, preventing the production of mature GPR65 mRNA. This methodological difference could contribute to phenotypic variations. (3) Hikiji et al. analyzed bone loss 4 weeks post‐OVX, when mice were approximately 3 months old. In contrast, our data indicate that female GPR65‐KO mice exhibit early bone loss trends at 2 months of age, with more pronounced effects by 6 months of age. To further investigate the clinical application potential of GPR65, we explored whether a GPR65 agonist has translational potential in the treatment of OP. We found that administration of the GPR65 agonist alone did not affect mouse growth, development, or bone metabolism (Figure S16). However, the GPR65 agonist effectively alleviated OVX‐induced OP in Balb/c mice (Figure 8), suggesting that GPR65‐mediated inhibition of bone loss is broadly consistent across models. These findings provide a robust foundation for future clinical studies and drug development.

As a proton‐sensing receptor, GPR65 senses the acidity and alkalinity of the extracellular environment and transmits corresponding signals within the cell. Interestingly, the extracellular pH environment plays a crucial regulatory role in cell activity and differentiation, and osteoclasts are involved in bone resorption by forming resorption lacunae on bone surfaces and secreting acidic substances into the lacunae (Komarova et al. 2005; Zhivodernikov et al. 2023). Moreover, extracellular acidic environments have been reported to promote osteoclast survival and bone resorption (Arnett 2008; Meghji et al. 2001; Xue et al. 2024). However, this study demonstrates that protons inhibit osteoclast differentiation via GPR65, and this discrepancy may exist for several reasons: (1) the pH values and treatment times used in the studies may be different; (2) the cells used in the research are different, with other studies focusing more on mature osteoclasts, while this study alters pH during the differentiation of osteoclast precursors into osteoclasts; (3) there may be other pathways regulated by extracellular acidic environments that modulate bone resorption, such as other proton‐sensing GPCRs (OGR1, GPR4, and G2A) or ion channels (including TRPV1 or ASICs) (Glitsch 2024); (4) the presence of various negative feedback and compensatory mechanisms; (5) whether there is a difference in the distribution of GPCRs, as seven‐transmembrane receptors, on the resorption lacuna side and basal side of osteoclasts, and whether the secretion of acidic substances by osteoclasts into the resorption lacunae causes cytoplasmic alkalization. To avoid cytoplasmic alkalization and its impact on their biological functions, osteoclasts need to reduce the cytoplasmic pH by inward chloride and outward bicarbonate anion exchange on the basal side, and whether this will lead to an increase in extracellular pH on the basal side of the osteoclast, thereby further promoting bone absorption. These issues still need further investigation.

Current studies have shown that GPR65 can respond to extracellular stimuli through Gαs, Gαq, Gα12/13, or Gαi, regulating a variety of physiological and pathological processes, including tumorigenesis, inflammatory bowel disease, pain, and inflammation (Chen et al. 2021; Neale et al. 2024; Zhang et al. 2023). Upon activation, GPR65 binds to Gαs to activate the AC/cAMP/PKA/CREB pathway, thereby regulating the transcription of target genes (Kottyan et al. 2009). If it binds to Gαi, it inhibits AC, thus inhibiting target gene transcription. The binding of GPR65 to Gαq activates the PLC‐β/PKC/MAPK pathway, which in turn regulates the expression of downstream genes (Zhang et al. 2023). Additionally, the activation of Gα12/13 regulates the transcription of target genes by activating RhoA. G proteins play a crucial role in bone development and remodeling processes. In human diseases, acquired mutations in Gαs can cause non‐MAS‐FD (non‐macrophage activation syndrome‐fibrous dysplasia), characterized by reduced bone ossification (Cong et al. 2019). In contrast, inactivating mutations in Gαs cause AHO (Albright hereditary osteodystrophy), characterized by short stature, ectopic ossification, and bone dysplasia (Cong et al. 2019; Long et al. 2007). Osteoblast‐specific knockout of Gαs, osteoblast‐specific overexpression of Gαi, and osteoblast‐specific overexpression of constitutively active Gαq all lead to reduced bone density and slowed bone remodeling (Cong et al. 2019; Ogata et al. 2011). Studies on the role of G proteins in osteoclast differentiation have confirmed that both Gαq and Gα12/13 are negative regulators of osteoclast formation and activity (Chakraborty et al. 2022; Luo et al. 2016; Wu et al. 2017). Our previous research confirmed the role of Gαq in mediating the regulatory effect of GPR65 on macrophage polarization (Zhang et al. 2023), leading us to hypothesize whether Gαq also mediates the inhibitory effect of GPR65 on osteoclast differentiation. Our findings indicate that either silencing or inhibiting Gαq significantly abrogates the inhibitory effect of GPR65 on osteoclast differentiation. The question then arises: which molecule does Gαq regulate to affect osteoclast differentiation? Our results indicate that the activation of GPR65 suppresses the expression and nuclear translocation of NFATc1, a key transcription factor in osteoclast differentiation, and it has been reported that GSK3β can regulate the nuclear translocation of NFATc1, thereby regulating the expression of target genes, including itself, playing a key role in T cell exhaustion and osteoclast differentiation. This suggests that GPR65, upon binding to Gαq, activates GSK3β, thereby inhibiting the expression of NFATc1 and promoting its nuclear export, inhibiting osteoclast differentiation (Figure S17). This is also supported by the observation that GSK3β silencing abolishes GPR65's inhibitory effect on osteoclast differentiation.

Although this study provides novel insights into the role of GPR65 in bone metabolism, several limitations should be acknowledged. For instance, while GPR65 is highly enriched in osteoclasts within bone tissue, suggesting that our global knockout model partially mimics an osteoclast‐specific conditional knockout model, a conditional overexpression model would provide more relevant insights. Moreover, a main limitation of this study is that in vitro osteoclast functional results are exclusively based on the quantification of TRAP‐positive, multinucleated cells as well as mRNA and protein expression analysis of osteoclast‐related genes. However, bone resorption activity assays on bovine bone slices showed no measurable resorptive activity (data not shown), necessitating further verification through optimized experiments. Additionally, the clinical relevance of GPR65 in human osteoporosis requires further investigation. In summary, our results confirm that GPR65 is a negative regulator of osteoclast differentiation with relative specificity in bone tissue, offering a promising molecular target for developing anti‐resorptive therapeutics.

## 4 Methods

### 4.1 Cell Culture

The mouse osteoclast precursor cells (murine immortalized macrophages) RAW264.7 cells were cultured in DMEM (Invitrogen) containing 1% penicillin and streptomycin (solarbio, Cat# P1400) and 10% FBS. The mouse bone marrow monocytes (BMMs) were isolated from the femur and tibia of C57BL/6 mice at 8 weeks of age, cultured in αMEM (Invitrogen, USA) containing 10% FBS (Gibco), 1% penicillin, and streptomycin (Hyclone, USA) and 10 ng/mL murine M‐CSF (Peprotech) for 5 days. Medium was changed every 2 days. Pre‐osteoblast cells MC3T3‐E1 clone 4 and primary human bone marrow mesenchymal stem cells (hMSCs) were cultured in αMEM (Invitrogen, USA) supplemented with 1% penicillin and streptomycin (Hyclone, USA) and 10% FBS (Gibco). To induce osteoclast differentiation, BMMs were treated with 25 ng/mL M‐CSF (PeproTech, Cat# 300‐25, USA) for 5 days, followed by 25 ng/mL M‐CSF (PeproTech, USA) together with 100 ng/mL RANKL (PeproTech, USA) for osteoclastogenesis. RAW 264.7 cells were induced with 100 ng/mL RANKL (PeproTech, USA). The medium was replaced every other day. All cells were cultured under conditions of 5% CO_2_ and 37°C. To induce osteoblastic mineralization, the cells were treated with osteogenic medium supplemented with 50 μg/mL of ascorbic acid (Sigma‐Aldrich), 5 mM glycerol 2‐phosphate (Sigma‐Aldrich) and 10 nM dexamethasone (Sigma‐Aldrich). Cells were treated with the GPR65 agonist BTB09089 (Maybridge, Cat# BTB09089; or Otava, Cat# 14900668; 30 μM), GPR65 inhibitor ZINC62678696 (Enamine, Cat# EN300‐261362; or Molport, Cat# molport‐019‐671‐510; 30 μM), and YM‐254890 (Focus Biomolecules, Cat# 10‐1590; 1 μM). For acidic exposure, DMEM or α‐MEM supplemented with 10% FBS was prepared by using biological buffer (either HEPES, HCl, or NaOH for pH 6.6, pH 8.2, and pH 7.2, respectively). Cells were treated with the designated pH, and the pH of the culture media was monitored by both observation of the medium color and measurement of the pH by precision test strips every 24 h. Adjustments were made as needed to maintain the desired pH throughout the experiment.

### 4.2 Tail Suspension (TS) Osteoporosis Model

Male C57BL/6 mice at 8 weeks of age were randomly divided into a TS group and a control group, with a minimum of 8 mice in the TS group. The middle and lower parts of the mouse tails were wrapped with a medical tape and fixed at an appropriate position on the cage cover. The hind limbs of the mice were suspended in the air, while the forelimbs were allowed to move freely within the cage, with normal access to food and water. The health status of the mice was monitored daily. Blood was collected via ocular puncture after 4 weeks of suspension. The hind limbs of the mice were collected, with the left hind limb fixed in neutral formalin and the right hind limb quickly frozen in liquid nitrogen before being stored at −80°C.

### 4.3 Ovariectomy (OVX) Osteoporosis Model

Female mice at 12 weeks of age were randomly assigned to an ovariectomized group (OVX group) or a control group, with a minimum of eight animals in the OVX group. Mice were anesthetized intraperitoneally with pentobarbital sodium based on body weight. After disinfection with iodine tincture, a 1 cm incision was made in the inguinal area on both sides to expose the milky white adipose mass encasing the ovaries. The ovaries were then excised, and the wounds were sutured and disinfected with iodine tincture. All mice were transferred to a warm environment for close observation. The incision sites on the back were disinfected daily with iodine tincture for 1 week following the operation. After 3 months, blood was collected via ocular puncture, and the mice from both the OVX group and the control group were euthanized by cervical dislocation. The hind limbs of the mice were harvested, with the left hind limb fixed in neutral formalin, and the right hind limb quickly frozen in liquid nitrogen before being stored at −80°C.

### 4.4 Animals In Vivo Study

All animal work was conducted according to the approved guidelines and approved by the Animal Care and Use Committee of Tianjin Medical University. Wild‐type (WT) mice and Gpr65 knockout (GPR65 KO) mice, which were generated using CRISPR‐Cas9 technology (Cyagen Biosciences, Suzhou, China) on a C57BL/6N background, were used in the TS‐, OVX‐, or aging‐induced osteoporosis models. In brief, the gRNAs sequence target the exon 2 of GPR65 were gRNA1: GGAGATTGGTCGGTGCAAATGGG; gRNA2: ATAACCCCTAAGAAGCACGCGGG. GPR65 KO mice were generated by crossing F0 heterozygous mice with wild‐type (WT) mice to produce F1 heterozygotes. Then, intercrossing F1 heterozygotes yielded F2 homozygous GPR65 KO mice and WT control mice. For TS and OVX models, 15 WT or GPR65 KO mice were separated into two groups randomly: WT and WT + TS/OVX or GPR65 KO and GPR65 KO + TS/OVX. For the aged model, 2‐, 6‐, and 12‐month‐old female WT and GPR65 KO mice were raised under standard animal housing environment. Moreover, 18 Balb/c mice at 8 weeks of age were purchased from Beijing HFK bioscience (Beijing, China) and separated into three groups randomly: DMSO, 10 mg/kg BTB09089 (the activator of GPR65), 20 mg/kg BTB09089. The GPR65 activator was administered for 8 weeks every 2 days. In addition, 24 Balb/c female mice at 8 weeks of age were purchased from Beijing HFK bioscience (Beijing, China) and separated into three groups randomly: DMSO, DMSO + OVX, and BTB09089 + OVX. The GPR65 activator (10 mg/kg) was administered 4 weeks after OVX operation every 2 days. All mice were euthanized to collect bilateral femurs and blood.

### 4.5 Microcomputed Tomography Analysis (Micro‐CT)

The left femurs and lumbar vertebrae dissected from the mice were fixed in 10% formalin for 48 h, then scanned and reconstructed with Skyscan1276 and NR econ. The scanning parameters were set as follows: current and voltage were set to 100 μA and 55 kV, respectively, with a resolution of 8 μm per pixel. Analysis was performed using CTAn software, and three‐dimensional reconstruction of the regions of interest (ROIs) was conducted using CTVol software. For the OVX model treated with GPR65 activator, the left femurs were scanned and analyzed with Inveon PET/SPECT/CT, Inveon Acquisition Workplace, COBRA_Exxim: licensed to Siemens, and Ineon Research Workplace. X‐ray current and voltage were set to 500 μA and 80 kV, with a resolution of 8.5 μm per pixel. The ROI selected for analysis was the distal femur beginning 0.1 mm below the growth plate and extending 100 layers proximally along the femur diaphysis. All trabecular bones were segmented for three‐dimensional reconstruction to calculate the parameters, including bone volume/total volume (BV/TV), trabecular number (Tb. N), trabecular thickness (Tb. Th), trabecular bone pattern factor (Tb. Pf), and structure model index (SMI). The cortical bone was analyzed in two dimensions and assessed for bone volume (BV) and mean cross‐sectional bone area (B. Ar). Mineral density was determined using a standard curve generated from a mineral density calibration phantom pair.

### 4.6 Enzyme‐Linked Immunosorbent Assay (ELISA)

Blood samples were harvested for measurements of bone turnover markers in serum. Serum levels of type I collagen C‐terminal telopeptide (CTX), tartrate‐resistant acid phosphatase (TRAP5b), procollagen type I N‐terminal propeptide (P1NP), and osteocalcin (OCN) were measured by commercially available ELISA kits according to manufacturers' instructions (Immunodiagnostic Systems, USA).

### 4.7 HE, TRAP Staining and Immunohistochemistry (IHC)

Mouse femur bone tissues were fixed in 4% paraformaldehyde solution for 2 days and decalcified in 0.5 M EDTA for 2–4 weeks, with EDTA changed twice a week. Subsequently, paraffin‐embedded samples were sliced into 6‐μm‐thick sections and then stained with Hematoxylineosin (HE), TRAP (Sigma, USA), or IHC. All sections were visualized by light microscopy. Antibody of GPR65 (AVIVA Systems Biology, OABF01813; 1:200) was used for IHC, and control IgG staining was included as a negative control. For osteoclast morphometric analyses, osteoclast number, osteoclast surface area, and eroded surface area were assessed by the OsteoMeasure Analysis System and ImageJ software according to the manufacturers' protocols. For cellular TRAP staining, cells were fixed with 2.5% glutaraldehyde in PBS for 30 min at room temperature and stained with the Leukocyte Acid Phosphatase Kit (Sigma) according to the manufacturer's instructions. TRAP‐positive cells with more than three nuclei were counted as mature osteoclasts.

### 4.8 Plasmid Construction and Cell Transfection

The full‐length cDNA of the murine GPR65 gene was amplified by PCR (Mouse Gpr65 BamH1 Forward: cgcggatccATGGCGATGAACAGCATGTG; Mouse Gpr65 Xho1 Reverse: ccgctcgagTGCCTCCACCTCTTAGTCTAT) and subsequently cloned into the pcDNA3.1(+) vector via the BamHI and XhoI restriction sites to obtain the overexpression plasmids (pcDNA3.1‐GPR65). The empty plasmid was used as control. Negative control vectors and pcDNA3.1‐GPR65 were transfected into cells at 60% confluence using Lipofectamine 2000/3000 (Invitrogen, USA, Cat# 11668019/L3000015). During transfection, we ensured that the cells were in good condition and used high‐purity, endotoxin‐free plasmid DNA (dissolved in sterile double‐distilled water) to enhance transfection efficiency. Non‐targeting siRNA (NC) and siRNAs targeting GPR65, Gαq and GSK3β were obtained from GenePharma Biological Technology (Shanghai, China). Cells were transfected with siRNAs at 40% confluence using Lipofectamine MAX (Invitrogen, USA, Cat# 13778150). After 24 to 48 h of culture, the knockdown and overexpression efficiency were analyzed by real‐time PCR. The siRNAs sequences are shown in Table S1.

### 4.9 qRT‐PCR Analysis

RNA isolation and quantitative real‐time PCR were performed as previously reported (Chen et al. 2022; Han et al. 2023; Ren et al. 2021; Zhang et al. 2023; Zhang et al. 2021). The relative level of each gene was normalized with the housekeeping gene GAPDH, and fold change was determined utilizing the $2^{-∆∆C_{t}}$ method. For cell‐by‐cell qPCR analysis, the data were analyzed based on GAPDH calibration and compared with BMM. The primer sequences for qRT‐PCR are provided in Table S2.

### 4.10 Western Blot

Protein isolation and western blot were performed as previously reported (Han et al. 2023; Zhang et al. 2017; Zhang et al. 2023). Antibodies used for western blot included NFATc1 (Rabbit polyclonal, Abcam, ab25916; RRID: AB_448901; 1:1000; Rabbit polyclonal, ABclonal, A1539, RRID: AB_2762296; 1:1000), MMP9 (Rabbit recombinant multiclonal, Abcam, ab283594, RRID: AB_3099506; 1:1000), CTSK (Rabbit monoclonal, Abcam, ab187647, RRID: AB_2891139; 1:1000), ATP6V0D2 (Mouse monoclonal, Abcam, ab236375; RRID: AB_3146157; 1:1000), GSK3β (Rabbit monoclonal, Cell Signaling Technology, 9315; RRID: AB_490890; 1:1000), Phospho‐GSK3β (Ser9) (Rabbit monoclonal, Cell Signaling Technology, 5558; RRID: AB_10013750; 1:1000; Rabbit polyclonal, ABclonal, AP0039, RRID: AB_2771151; 1:1000), Gαq (Rabbit monoclonal, Abcam, ab210004; RRID:AB_2934134; 1:1000), GAPDH (mouse monoclonal, Abcam, ab8245; RRID: AB_2107448; 1:8000). GAPDH was used as an internal control.

### 4.11 Confocal Microscopy

RAW264.7 cells were seeded in glass coverslips directly, then induced with 50 ng/mL RANKL (PeproTech, USA) for 3 days and transfected with or without Gαq or GSK3β siRNAs using lipofectamine MAX. Antibodies used for confocal microscopy included NFATc1 (1:100, Abcam, USA, ab25916) and an irrelevant isotype rabbit IgG. Cells were incubated with the proper fluorescent secondary antibody (Alexa Fluor 647, Invitrogen, USA) at 37°C for 1 h away from light. DAPI (10 μg/mL, Sigma, USA, Cat# B2883) was used to stain the nuclei.

### 4.12 Sequencing Dataset Analysis

Three osteoclastogenesis‐related datasets from the GEO database were selected: (1) The GSE72047 dataset, which comprises samples of mouse bone marrow‐derived macrophages (BMMs) induced or not induced with macrophage colony‐stimulating factor (M‐CSF) and receptor activator of nuclear factor kappa‐B ligand (RANKL); (2) The GSE74847 dataset, encompassing samples of RAW264.7 cells induced or not induced with 50 ng/mL RANKL; (3) The GSE225974 dataset, which includes samples of human osteoclast‐like cells (OC) and their precursors, peripheral blood mononuclear cells (PBMC). Data quality control analysis and differential analysis were performed using the R programming language. The criteria for selecting differentially expressed genes were an absolute value of Log2(FC) greater than 1 and a *p*adj value less than 0.05.

### 4.13 Statistical Analysis

Data were expressed as mean ± SEM with at least three independent experiments. All statistical analyses were conducted using GraphPad7. Statistical analysis was conducted using either one‐way analysis of variance (more than two groups) or Student's *t* test (two group comparison), and *p* < 0.05 indicates a significant difference.

## Author Contributions

Conceptualization, W.H. and K.Z.; methodology, K.Z., Y.L., Y.R., Y.H.; investigation, Y.L., K.Z., Y.R., Y.H., J.W., X.L., K.G., Y.Y., Z.S., and L.Z.; writing – original draft, K.Z.; writing – review and editing, W.H., and K.Z.; funding acquisition, W.H., K.Z., and Z.S.; supervision, W.H., and K.Z.

## Conflicts of Interest

The authors declare no conflicts of interest.

## Supporting information


**Figure S1:** Identification of GPR65 in osteoclasts' differentiation, related to Figure 1.
**Figure S2:** Deletion of Gpr65 results in age‐related bone loss and OP, related to Figure 2.
**Figure S3:** Deletion of Gpr65 exacerbates TS‐induced OP, related to Figure 3.
**Figure S4:** Deletion of Gpr65 exacerbates OVX‐induced OP, related to Figure 4.
**Figure S5:** GPR65 inhibits osteoclast differentiation of RAW264.7 cells, related to Figure 5.
**Figure S6:** GPR65 does not regulate osteoblast differentiation, related to Figure 5.
**Figure S7:** Acidic extracellular environment inhibits BMM osteoclast differentiation through GPR65, related to Figure 5.
**Figure S8:** Acidic extracellular environment inhibits RAW264.7 cells' osteoclast differentiation through GPR65.
**Figure S9:** Effect of GPR65 exogenous agonist and inhibitor on osteoclast differentiation of BMM.
**Figure S10:** Effect of GPR65 exogenous agonist and inhibitor on osteoclast differentiation of RAW264.7 cells.
**Figure S11:** Endogenous ligand of GPR65 inhibits osteoclast differentiation through Gαq, related to Figure 6.
**Figure S12:** GPR65 agonist inhibits osteoclast differentiation through Gαq, related to Figure 6.
**Figure S13:** GPR65 inhibits GSK3β phosphorylation, related to Figure 7.
**Figure S14:** GPR65 inhibits osteoclast differentiation via GSK3β, related to Figure 7.
**Figure S15:** GPR65 suppresses the expression and nuclear translocation of NFATc1 via Gαq and GSK3β, related to Figure 7.
**Figure S16:** Intraperitoneal injection of BTB09089 does not affect bone metabolism, related to Figure 8.
**Figure S17:** Schematic diagram illustrating the function and mechanism of GPR65 in regulating osteoclast differentiation and osteoporosis.
**Table S1:** siRNA sequences.
**Table S2:** Quantitative real‐time reverse transcription‐polymerase chain reaction (qRT‐PCR) primers for the analysis of transcript levels.

## Data Availability

All the data supporting the findings of this study are available within the article and its Supporting Information files or from the corresponding author upon reasonable request.
