# Supplementary material for: GPR65 Functions as a Key Factor of Bone Aging and a Novel Therapeutic Target for Osteoporosis
Source: Aging Cell. 2025 Sep 2;24(10):e70212. doi: 10.1111/acel.70212 (PMC12507399; doi:10.1111/acel.70212)
Supplement: Supplementary file 1 — Figure S1: Identification of GPR65 in osteoclasts' differentiation, related to Figure 1. Figure S2: Deletion of Gpr65 results in age‐related bone loss and OP, related to Figure 2. Figure S3: Deletion of Gpr65 exacerbates TS‐induced OP, related to Figure 3. Figure S4: Deletion of Gpr65 exacerbates OVX‐induced OP, related to Figure 4. Figure S5: GPR65 inhibits osteoclast differentiation of RAW264.7 cells, related to Figure 5. Figure S6: GPR65 does not regulate osteoblast differentiation, related to Figure 5. Figure S7: Acidic extracellular environment inhibits BMM osteoclast differentiation through GPR65, related to Figure 5. Figure S8: Acidic extracellular environment inhibits RAW264.7 cells' osteoclast differentiation through GPR65. Figure S9: Effect of GPR65 exogenous agonist and inhibitor on osteoclast differentiation of BMM. Figure S10: Effect of GPR65 exogenous agonist and inhibitor on osteoclast differentiation of RAW264.7 cells. Figure S11: Endogenous ligand of GPR65 inhibits osteoclast differentiation through Gαq, related to Figure 6. Figure S12: GPR65 agonist inhibits osteoclast differentiation through Gαq, related to Figure 6. Figure S13: GPR65 inhibits GSK3β phosphorylation, related to Figure 7. Figure S14: GPR65 inhibits osteoclast differentiation via GSK3β, related to Figure 7. Figure S15: GPR65 suppresses the expression and nuclear translocation of NFATc1 via Gαq and GSK3β, related to Figure 7. Figure S16: Intraperitoneal injection of BTB09089 does not affect bone metabolism, related to Figure 8. Figure S17: Schematic diagram illustrating the function and mechanism of GPR65 in regulating osteoclast differentiation and osteoporosis. Table S1: siRNA sequences. Table S2: Quantitative real‐time reverse transcription‐polymerase chain reaction (qRT‐PCR) primers for the analysis of transcript levels. [file ACEL-24-e70212-s001.docx]

**Supplemental information**

**Supplementary Figures and Figure Legends**

**
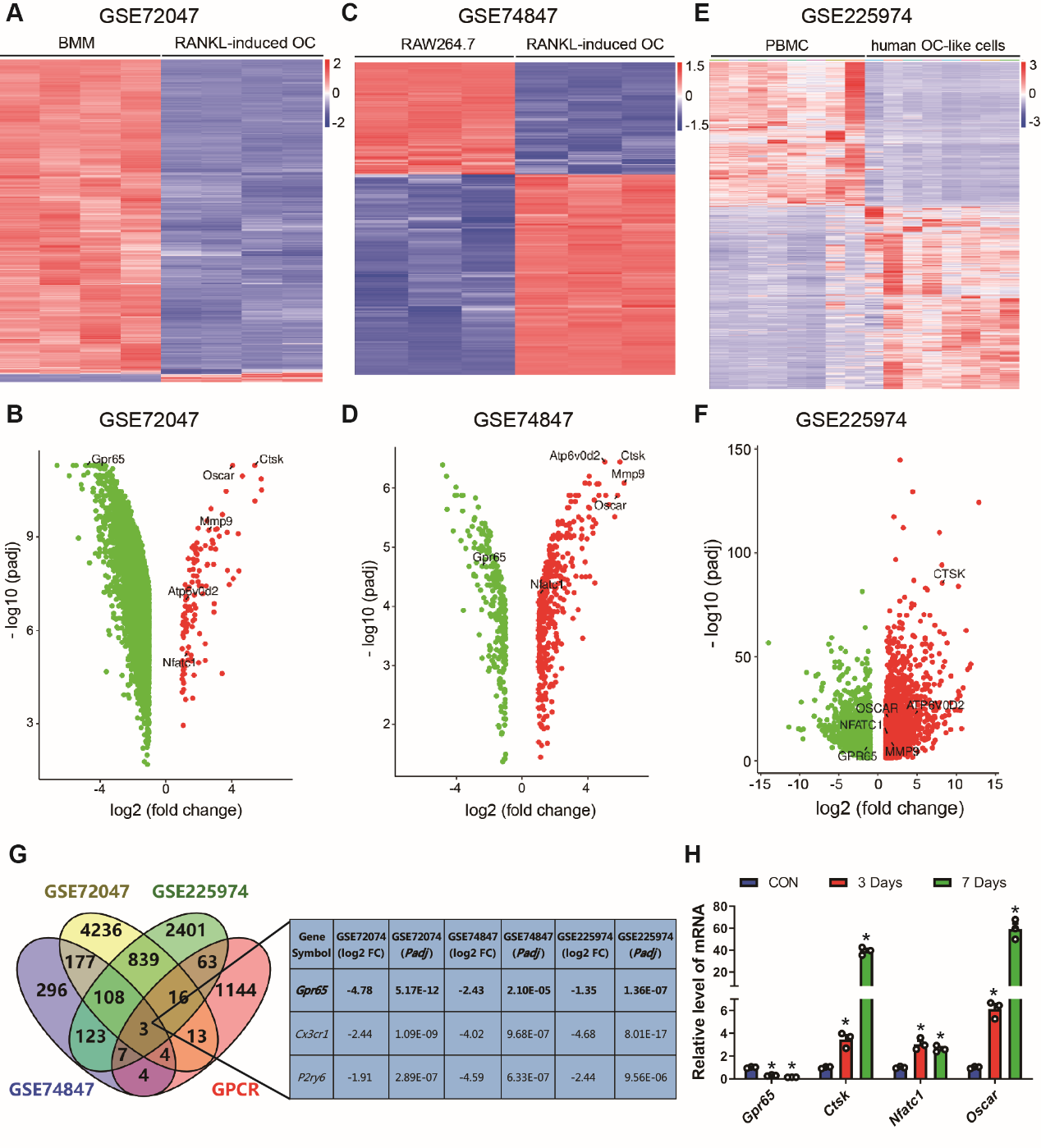
**

**Figure S1. Identification of GPR65 in osteoclasts differentiation****, related to Figure 1**

(A-F) Hierarchical cluster and volcano map analysis of differentially expressed mRNAs in osteoclasts differentiation, acquired from the GEO database: GSE72047, GSE74847, and GSE225974; bright red, up-regulation; bright green, down-regulation. (G) The Venn diagram of differentially expressed mRNAs in GSE72047, GSE74847, GSE225974, and the GPCRs gene set. (H) qRT-PCR analysis of *Gpr65, Ctsk, Nfatc1* and *Oscar* in RAW264.7 cells cultured in osteoclast differentiation medium for 3 or 7 days (n = 3). ^*^*p* < 0.05 for vs CON.


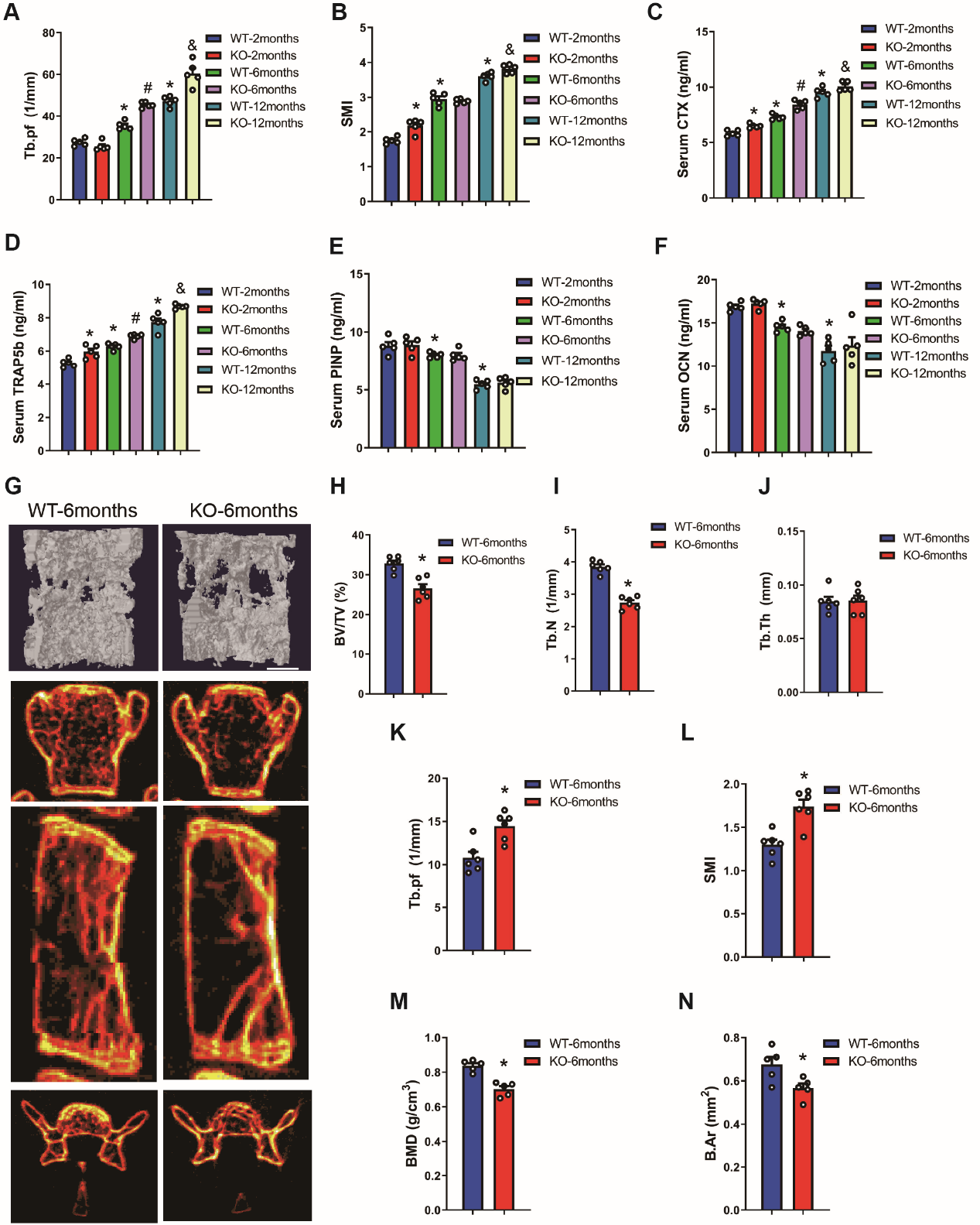


**Figure S2. Deletion of Gpr65 results in age-related bone loss and OP, related to Figure 2**

(A, B) Micro-CT analysis of distal femurs from 2-month-old, 6-month-old and 12-month-old female WT and GPR65 KO mice for trabecular bone pattern factor (Tb. Pf) and structure model index (SMI) (n = 5). (C-F) Serum CTX, TRAP5b, PINP and OCN levels in 2-month-old, 6-month-old and 12-month-old female WT and GPR65 KO mice (n = 5). (G-L) Representative 2D and 3D images, as well as micro-CT analysis, of lumbar vertebral trabecular bone L5 from 6-month-old female WT and GPR65 KO mice for BV/TV, Tb.N, Tb.Th, Tb. Pf and SMI (n = 6). (M, N) Micro-CT analysis of femoral cortical bone from 6-month-old female WT and GPR65 KO mice for Bone mineral density (BMD) and mean total cross-sectional thickness bone area (B.Ar) (n = 5). ^*^*p* < 0.05 for vs WT-2months. ^#^*p* < 0.05 for vs WT-6months. ^&^*p* < 0.05 for vs WT-12months.

**
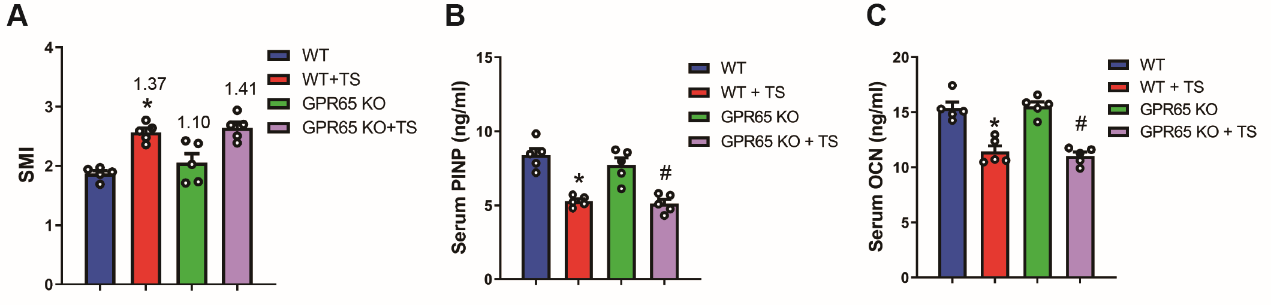
**

**Figure S3. Deletion of Gpr65 exacerbates TS-induced OP, related to Figure 3**

(A) Micro-CT analysis of distal femurs from WT and GPR65 KO mice with or without TS treatment for SMI, values in the figure represent ratios relative to WT (n = 5). (B, C) Serum PINP and OCN levels in WT and GPR65 KO mice with or without TS treatment (n = 5). ^*^*p* < 0.05 for vs WT. ^#^*p* < 0.05 for vs WT + TS.

**
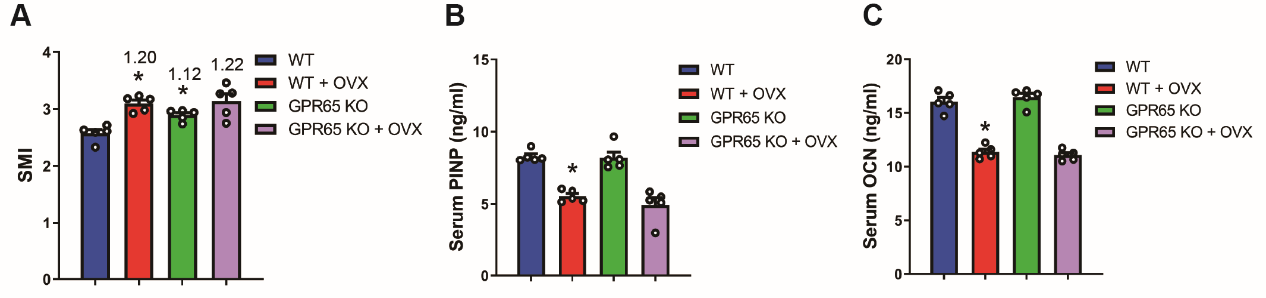
**

**Figure S4. Deletion of Gpr65 exacerbates OVX-induced OP, related to Figure 4**

(A) Micro-CT analysis of distal femurs from WT and GPR65 KO mice with or without OVX treatment for SMI, values in the figure represent ratios relative to WT (n = 5). (B, C) Serum PINP and OCN levels in WT and GPR65 KO mice with or without OVX treatment (n = 5). ^*^*p* < 0.05 for vs WT. ^#^*p* < 0.05 for vs WT + OVX.

**
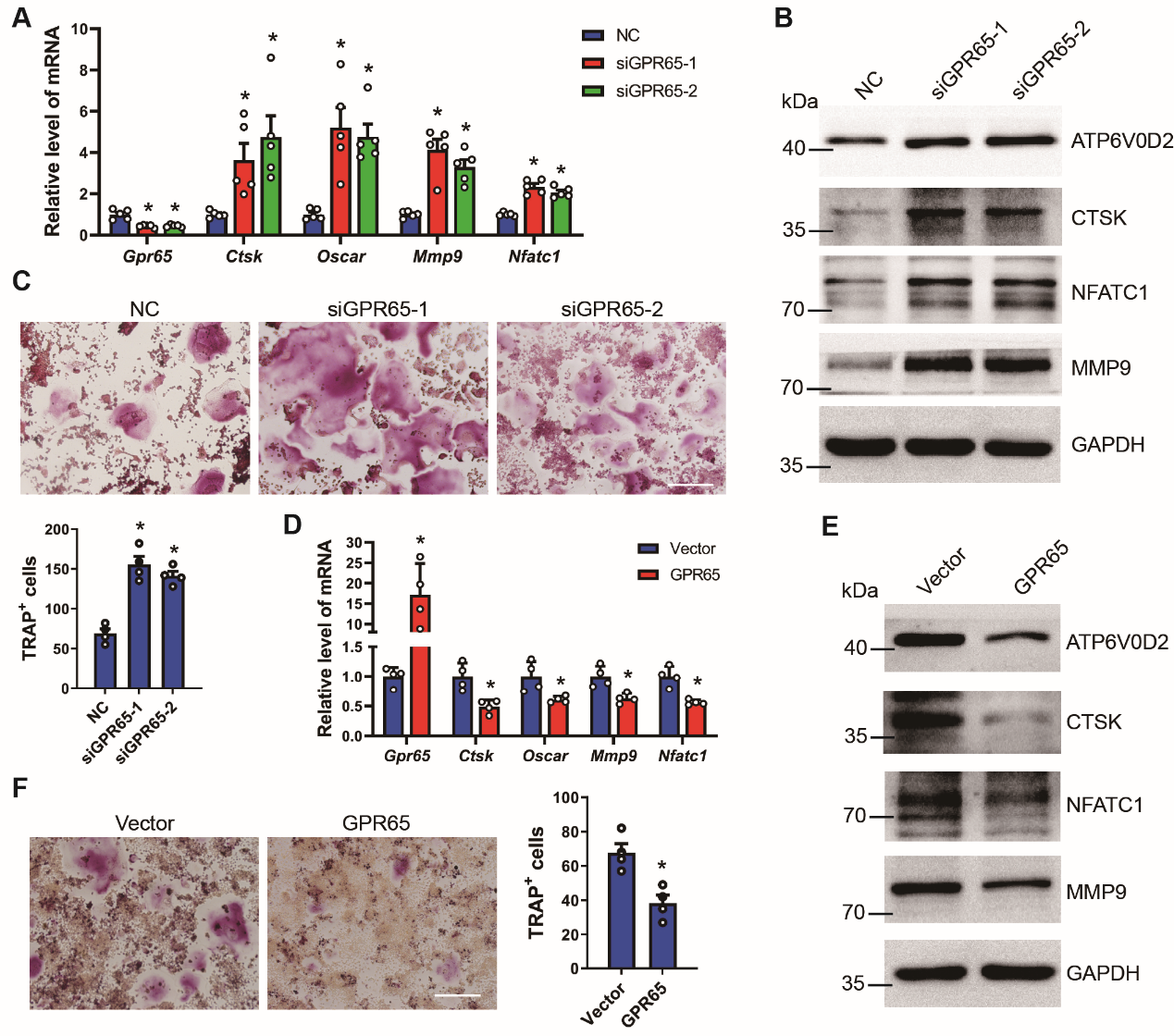
**

**Figure S5. GPR65 inhibits osteoclast differentiation of RAW264.7 cells, related to Figure 5**

(A-C) RAW264.7 cells were transfected with GPR65-siRNAs or negative control siRNA and simultaneously cultured in osteoclast differentiation medium (3 days for qRT-PCR and Western blot; 5 days for TRAP staining). qRT-PCR analysis of the expression of *Gpr65, Ctsk, Oscar, Mmp9* and *Nfatc1* (A) (n = 5); The protein level of ATP6V0D2, CTSK, NFATc1 and MMP9 was determined by western blot (B); GAPDH was used as an internal control. Representative TRAP staining images of osteoclasts (C); scale bar, 200 μm. Quantifications are shown in the bottom panel (n = 4). (D-F) RAW264.7 cells were transfected with pcDNA3.1-GPR65 or pcDNA3.1 and simultaneously cultured in osteoclast differentiation medium (3 days for qRT-PCR and Western blot; 5 days for TRAP staining). qRT-PCR analysis of the expression of *Gpr65, Ctsk, Oscar, Mmp9* and *Nfatc1* (D) (n = 4); The protein level of ATP6V0D2, CTSK, NFATc1 and MMP9 was determined by western blot (E); GAPDH was used as an internal control. Representative TRAP staining images of osteoclasts (F); scale bar, 200 μm. Quantifications are shown in the right panel (n = 4). ^*^*p* < 0.05 for vs NC or Vector.

**
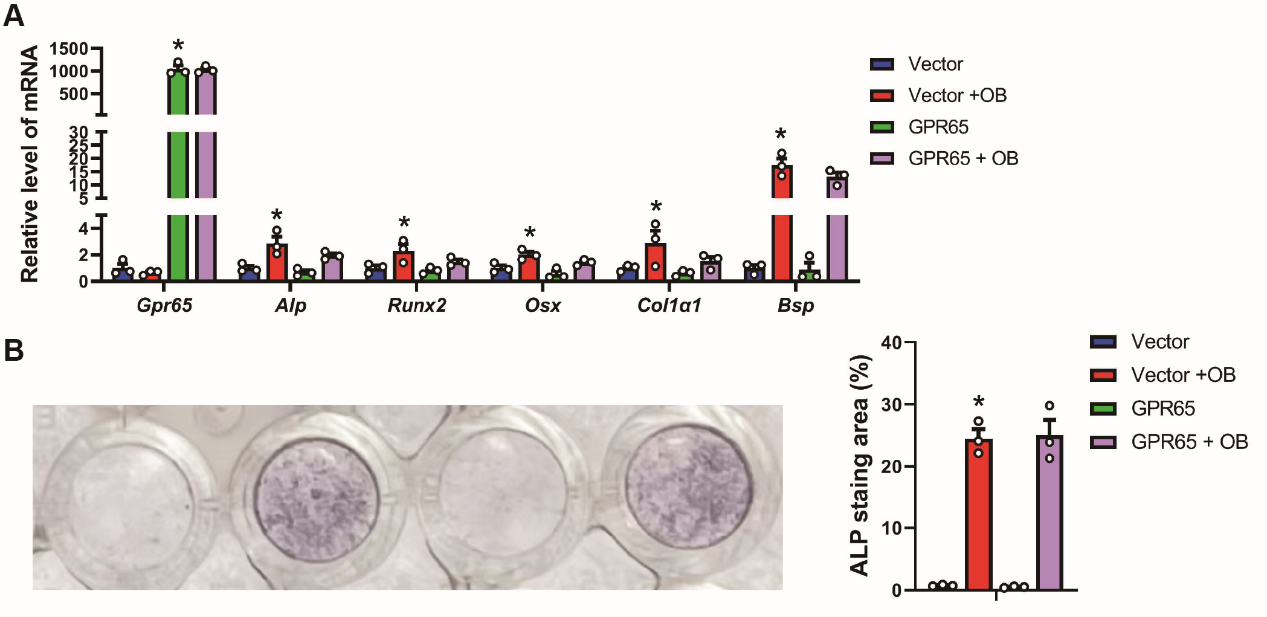
**

**Figure S6. GPR65 does not regulate osteoblast differentiation, related to Figure 5**

(A, B) MC3T3-e1 cells were transfected with pcDNA3.1-GPR65 or pcDNA3.1 and simultaneously cultured with or without osteoblast differentiation medium. qRT-PCR analysis of the expression of *Gpr65, Alp, Runx2, Osx, Col1α1* and *Bsp* (A) (n = 3); Representative ALP staining images (B). Quantifications are shown in the right panel (n = 3). ^*^*p* < 0.05 for vs Vector.

**
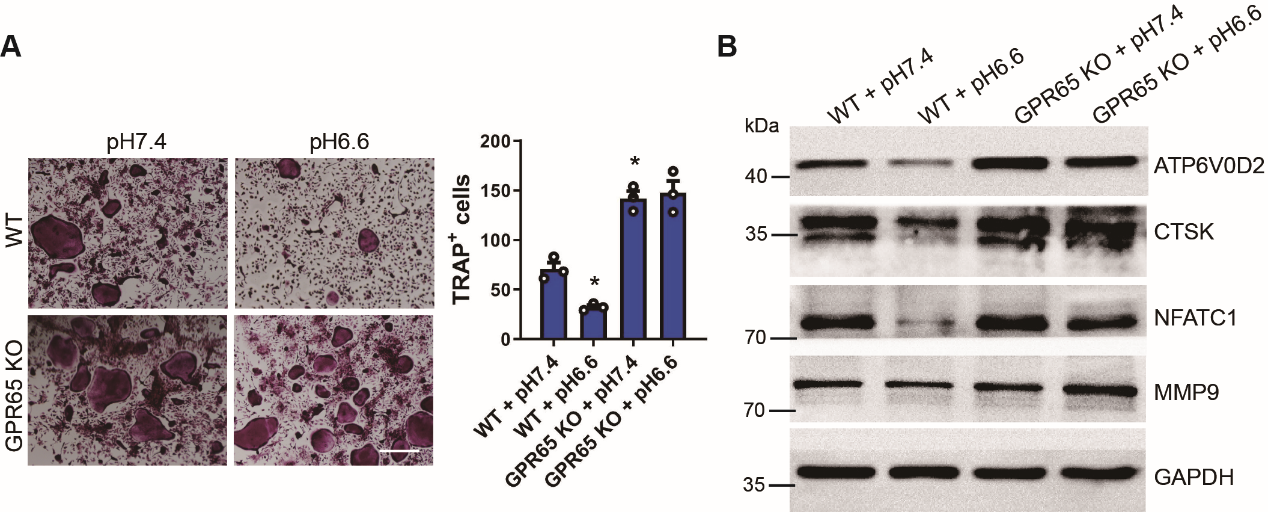
**

**Figure S7. Acidic extracellular environment inhibits BMM osteoclast differentiation through GPR65, related to Figure 5**

(A, B) WT and GPR65 KO BMM were incubated in pH 7.4 and pH 6.6, and simultaneously cultured in osteoclast differentiation medium for 8 days. Representative TRAP staining images of osteoclasts (A); scale bar, 200 μm. Quantifications are shown in the right panel (n = 3). The protein level of ATP6V0D2, CTSK, NFATc1 and MMP9 was determined by western blot (B); GAPDH was used as an internal control. ^*^*p* < 0.05 for vs WT + pH7.4.

**
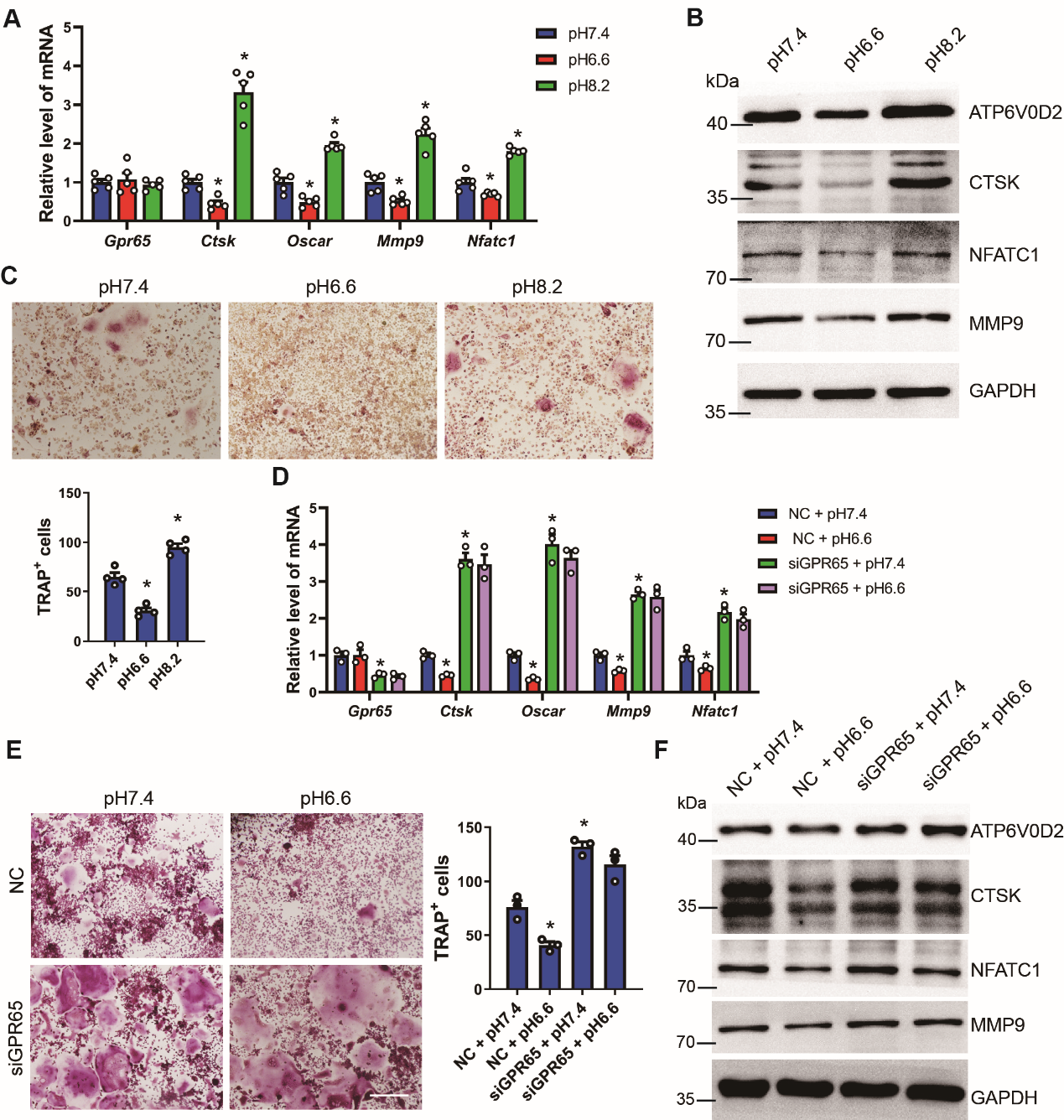
**

**Figure S8. Acidic extracellular environment inhibits RAW264.7 cells osteoclast differentiation through GPR65**

(A-C) RAW264.7 cells were incubated in the physiological pH 7.4, the acidic pH 6.6 or the alkalic pH 8.2 and simultaneously cultured in osteoclast differentiation medium (3 days for qRT-PCR and Western blot; 5 days for TRAP staining). qRT-PCR analysis of the expression of *Gpr65, Ctsk, Oscar, Mmp9* and *Nfatc1* (A) (n = 5); The protein level of ATP6V0D2, CTSK, NFATc1 and MMP9 was determined by western blot (B); GAPDH was used as an internal control. Representative TRAP staining images of osteoclasts (C); scale bar, 200 μm. Quantifications are shown in the bottom panel (n = 4). (D-F) Control and GPR65-silenced RAW264.7 cells were incubated in pH 7.4 and pH 6.6, and simultaneously cultured in osteoclast differentiation medium (3 days for qRT-PCR and Western blot; 5 days for TRAP staining). qRT-PCR analysis of the expression of *Gpr65, Ctsk, Oscar, Mmp9* and *Nfatc1* (D) (n = 3); Representative TRAP staining images of osteoclasts (E); scale bar, 200 μm. Quantifications are shown in the right panel (n = 3). The protein level of ATP6V0D2, CTSK, NFATc1 and MMP9 was determined by western blot (F); GAPDH was used as an internal control. ^*^*p* < 0.05 for vs pH7.4 or NC + pH7.4.

**
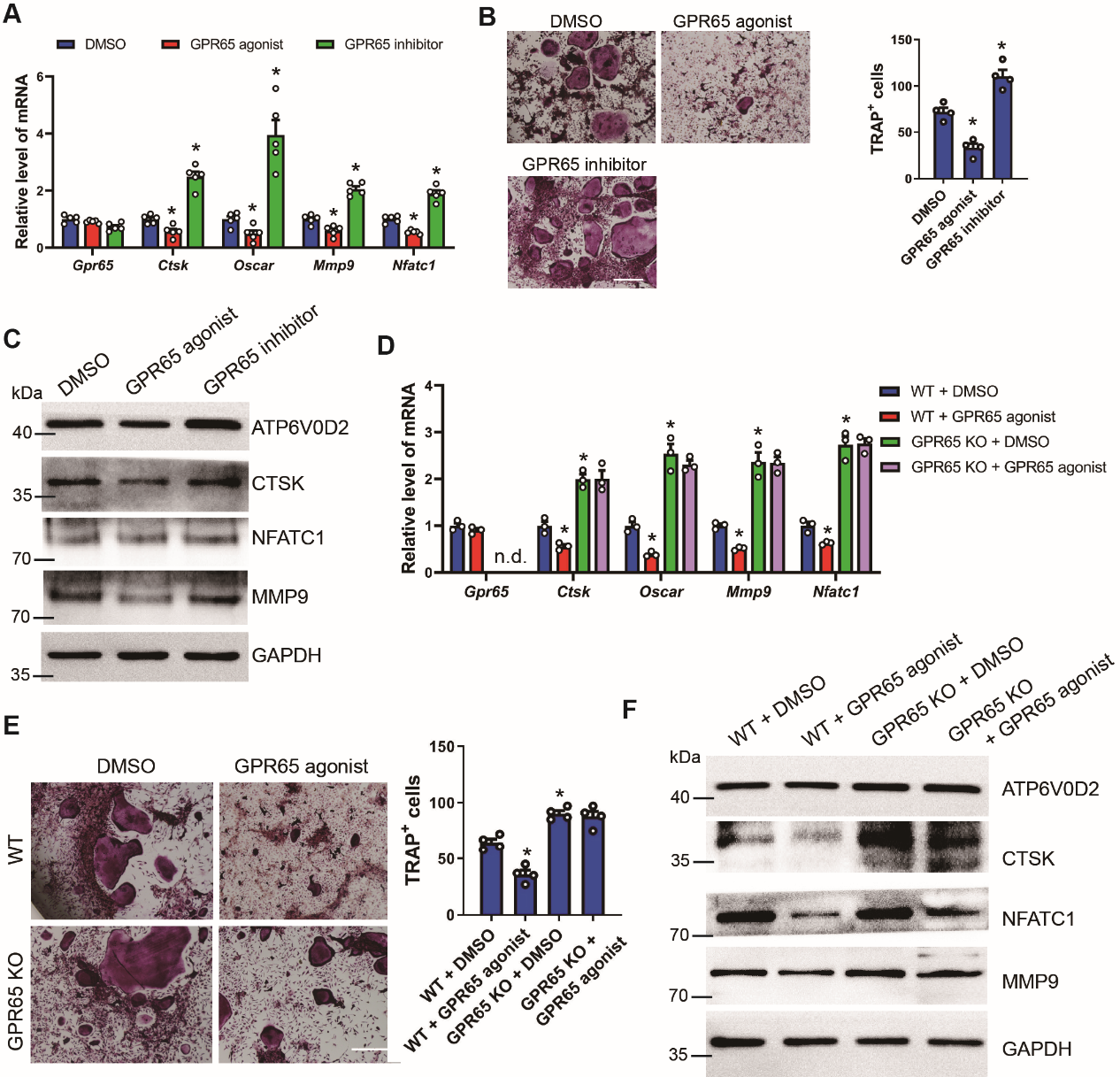
**

**Figure S9. Effect of GPR65 exogenous agonist and inhibitor on osteoclast differentiation of BMM**

(A-C) BMMs were treated with DMSO, 30 μM GPR65 exogenous agonist BTB09089 or GPR65 exogenous inhibitor ZINC62678696, and simultaneously cultured in osteoclast differentiation medium for 8 days. qRT-PCR analysis of the expression of *Gpr65, Ctsk, Oscar, Mmp9* and *Nfatc1* (A) (n = 5); Representative TRAP staining images of osteoclasts (B) (n = 4); scale bar, 200 μm. Quantifications are shown in the right panel. The protein level of ATP6V0D2, CTSK, NFATc1 and MMP9 was determined by western blot (C); GAPDH was used as an internal control. (D-F) WT and GPR65 KO BMM were treated with DMSO or 30 μM BTB09089, and simultaneously cultured in osteoclast differentiation medium for 8 days. qRT-PCR analysis of the expression of *Gpr65, Ctsk, Oscar, Mmp9* and *Nfatc1* (D) (n = 3); Representative TRAP staining images of osteoclasts (E); scale bar, 200 μm. Quantifications are shown in the right panel (n = 4). The protein level of ATP6V0D2, CTSK, NFATc1 and MMP9 was determined by western blot (F); GAPDH was used as an internal control. ^*^*p* < 0.05 for vs DMSO or WT + DMSO.

**
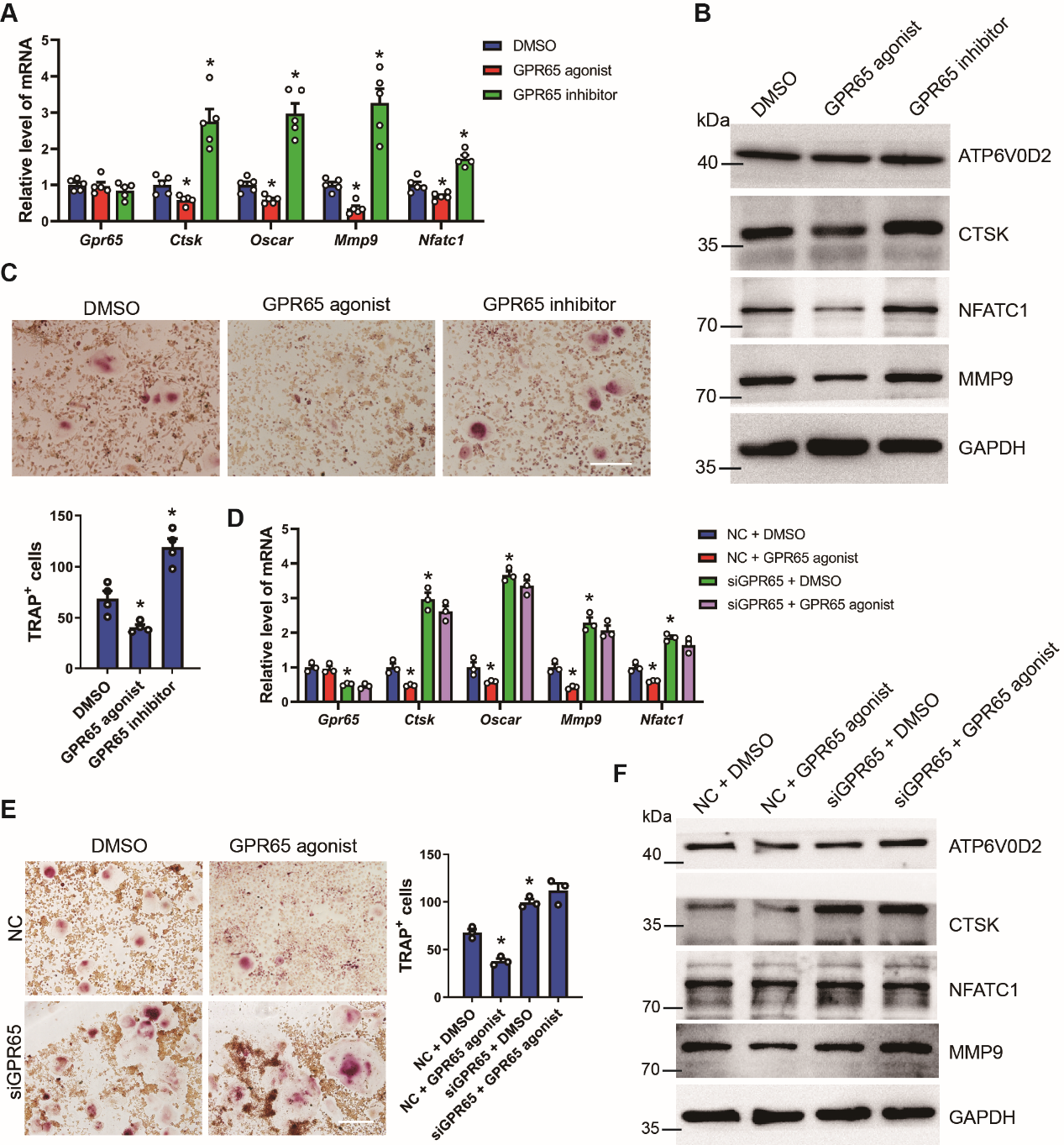
**

**Figure S10. Effect of GPR65 exogenous agonist and inhibitor on osteoclast differentiation of RAW264.7 cell**

(A-C) RAW264.7 cells were treated with DMSO, 30 μM BTB09089 or ZINC62678696, and simultaneously cultured in osteoclast differentiation medium (3 days for qRT-PCR and Western blot; 5 days for TRAP staining). qRT-PCR analysis of the expression of *Gpr65, Ctsk, Oscar, Mmp9* and *Nfatc1* (A) (n = 5); The protein level of ATP6V0D2, CTSK, NFATc1 and MMP9 was determined by western blot (B); GAPDH was used as an internal control. Representative TRAP staining images of osteoclasts (C); scale bar, 200 μm. Quantifications are shown in the bottom panel (n = 3). (D-F) Control and GPR65-silenced RAW264.7 cells were treated with DMSO or 30 μM BTB09089, and simultaneously cultured in osteoclast differentiation medium. qRT-PCR analysis of the expression of *Gpr65, Ctsk, Oscar, Mmp9* and *Nfatc1* (D) (n = 3); Representative TRAP staining images of osteoclasts (E); scale bar, 200 μm. Quantifications are shown in the right panel (n = 3). The protein level of ATP6V0D2, CTSK, NFATc1 and MMP9 was determined by western blot (F); GAPDH was used as an internal control. ^*^*p* < 0.05 for vs DMSO or NC + DMSO.

**
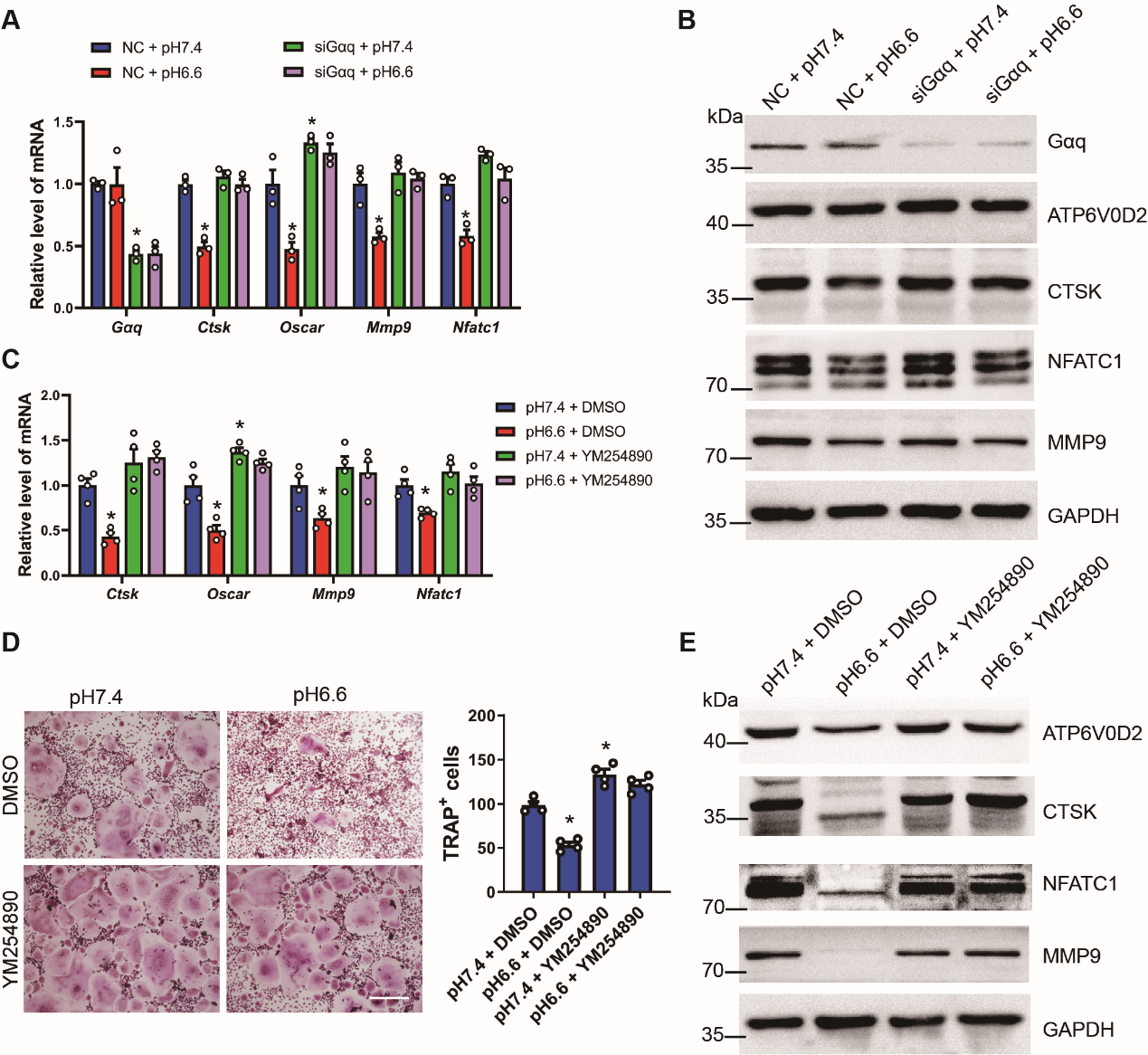
**

**Figure S11. The endogenous ligand of GPR65 inhibits osteoclast differentiation through Gαq, related to Figure 6**

(A, B) pH7.4- or pH6.6- treated RAW264.7 cells were transfected with Gαq-siRNA or negative control, and simultaneously cultured in osteoclast differentiation medium for 3 days. qRT-PCR analysis of the expression of *Gαq, Ctsk, Oscar, Mmp9* and *Nfatc1* (A) (n = 3); The protein level of ATP6V0D2, CTSK, NFATc1 and MMP9 was determined by western blot (B). (C-E) pH7.4- or pH6.6- treated RAW264.7 cells were treated with or without 1 μM Gαq inhibitor YM254890, and simultaneously cultured in osteoclast differentiation medium (3 days for qRT-PCR and Western blot; 5 days for TRAP staining). qRT-PCR analysis of the expression of *Ctsk, Oscar, Mmp9* and *Nfatc1* (C) (n = 4); Representative TRAP staining images of osteoclasts (D); scale bar, 200 μm. Quantifications are shown in the right panel (n = 4). The protein level of ATP6V0D2, CTSK, NFATc1 and MMP9 was determined by western blot (E); GAPDH was used as an internal control. ^*^*p* < 0.05 for vs pH7.4 + NC or pH7.4 + DMSO.

**
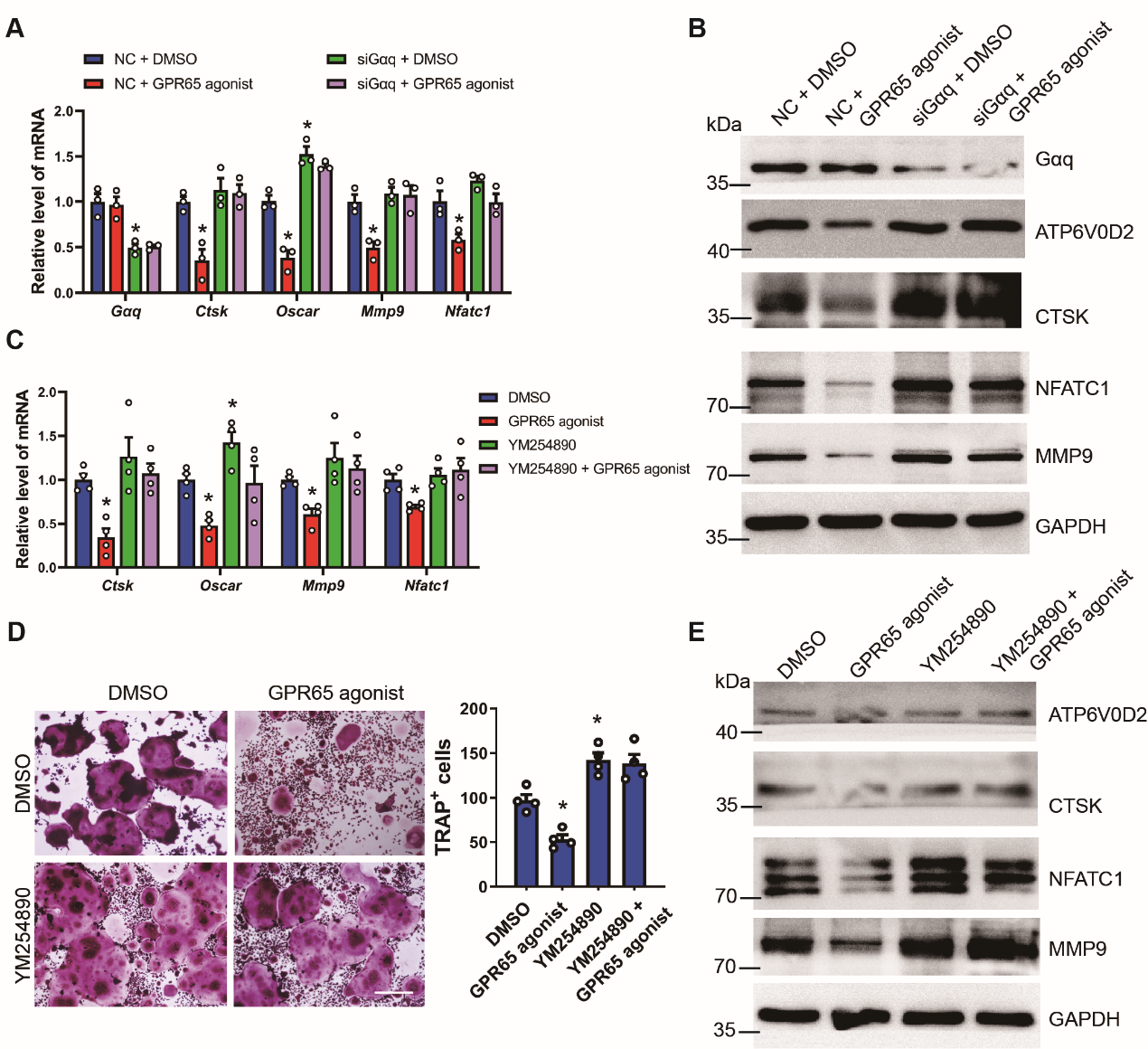
**

**Figure S12. GPR65 agonist inhibits osteoclast differentiation through Gαq, related to Figure 6**

(A, B) DMSO- or BTB09089- treated RAW264.7 cells were transfected with Gαq-siRNA or negative control, and simultaneously cultured in osteoclast differentiation medium for 3 days. qRT-PCR analysis of the expression of *Gαq, Ctsk, Oscar, Mmp9* and *Nfatc1* (A) (n = 3); The protein level of ATP6V0D2, CTSK, NFATc1 and MMP9 was determined by western blot (B); GAPDH was used as an internal control. (C-E) DMSO- or BTB09089- treated RAW264.7 cells were treated with or without 1 μM Gαq inhibitor YM254890, and simultaneously cultured in osteoclast differentiation medium (3 days for qRT-PCR and Western blot; 5 days for TRAP staining). qRT-PCR analysis of the expression of *Ctsk, Oscar, Mmp9* and *Nfatc1* (C) (n = 4); Representative TRAP staining images of osteoclasts (D); scale bar, 200 μm. Quantifications are shown in the right panel (n = 4). The protein level of ATP6V0D2, CTSK, NFATc1 and MMP9 was determined by western blot (E); GAPDH was used as an internal control. ^*^*p* < 0.05 for vs p NC +DMSO or DMSO.

**
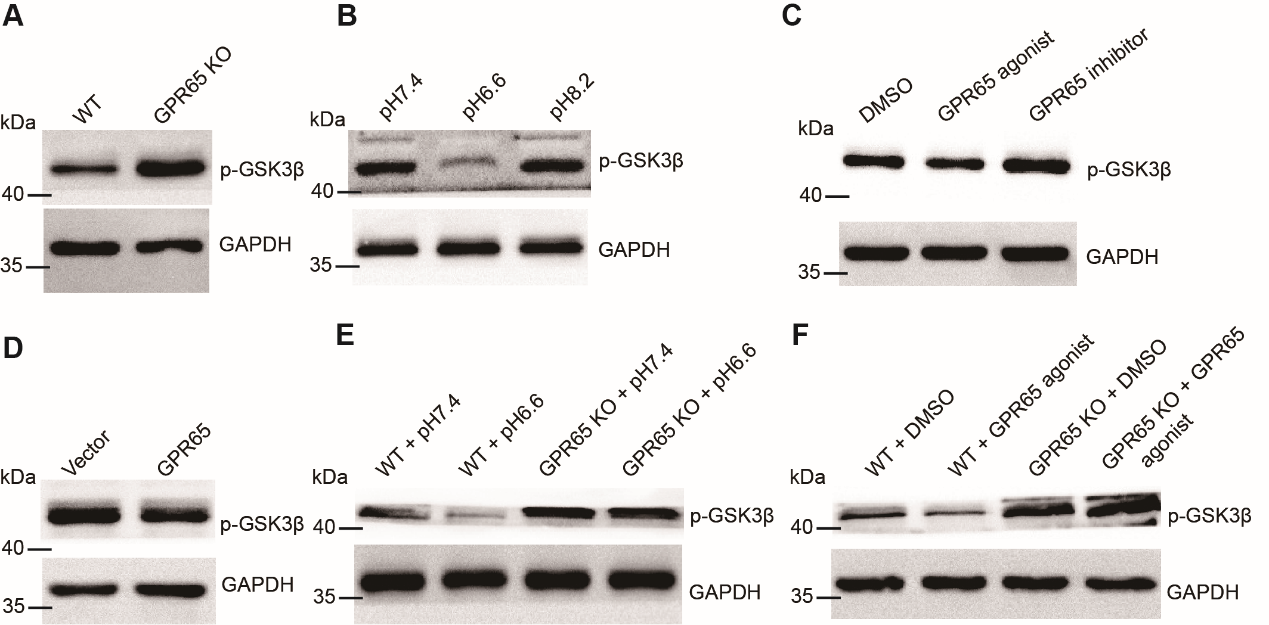
**

**Figure S13. GPR65 inhibits GSK3β phosphorylation, related to Figure 7**

(A-F) The protein level of phos-GSK3β and total GSK3β in GPR65-knockouted, GPR65-over-expressed, various pH-treated or GPR65 agonist/inhibitor-treated BMM cultured in osteoclast differentiation medium for 8 days simultaneously was determined by western blot; GAPDH was used as an internal control.

**
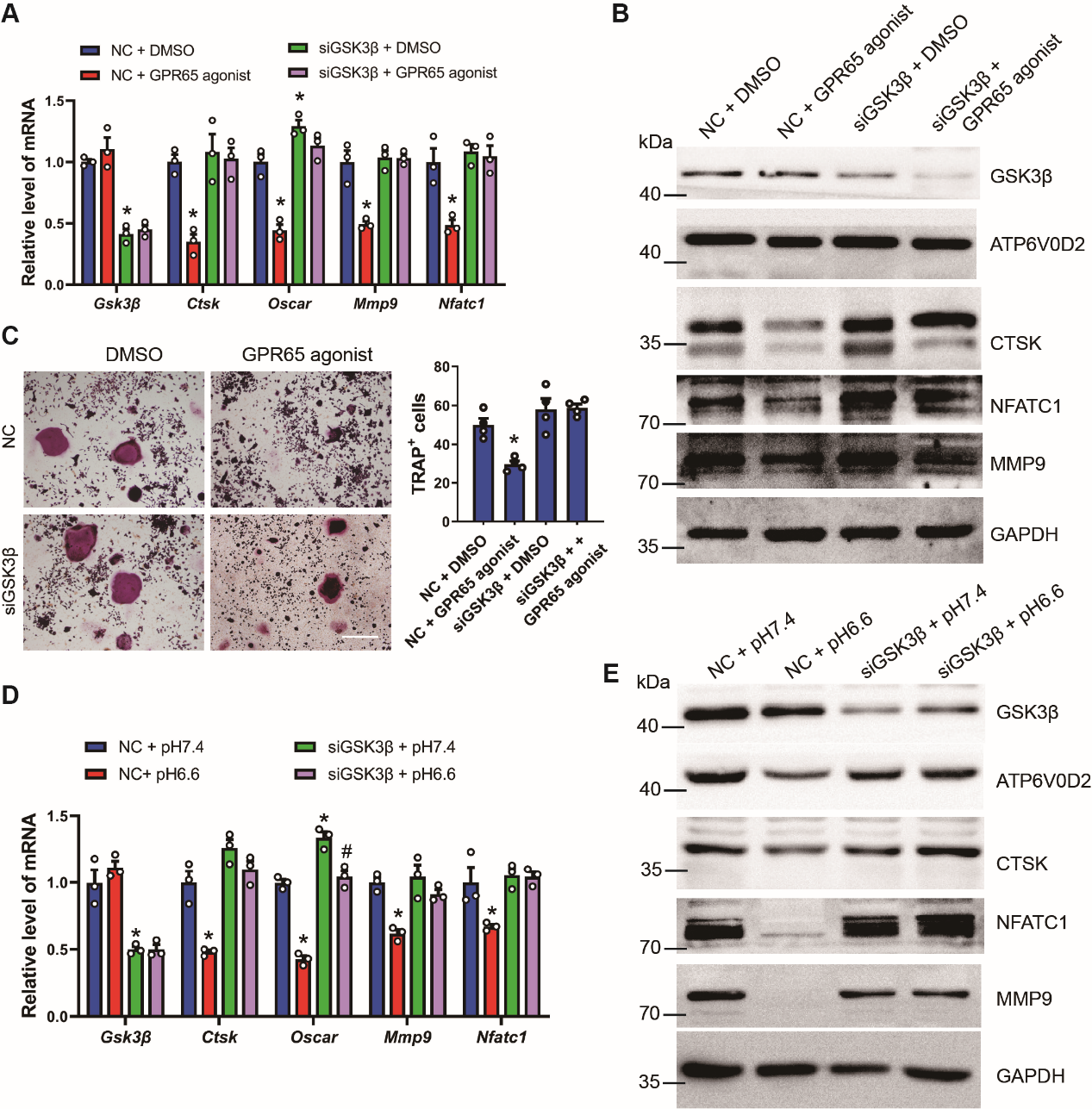
**

**Figure S14. GPR65 inhibits osteoclast differentiation via GSK3β, related to Figure 7**

(A-C) DMSO- or BTB09089- treated RAW264.7 cells were transfected with GSK3β-siRNA or negative control, and simultaneously cultured in osteoclast differentiation medium (3 days for qRT-PCR and Western blot; 5 days for TRAP staining). qRT-PCR analysis of the expression of *Gsk3β, Ctsk, Oscar, Mmp9* and *Nfatc1* (A) (n = 3); The protein level of GSK3β, ATP6V0D2, CTSK, NFATc1 and MMP9 was determined by western blot (B); GAPDH was used as an internal control. Representative TRAP staining images of osteoclasts (C); scale bar, 200 μm. Quantifications are shown in the right panel (n = 4). (D, E) pH7.4- or pH6.6- treated RAW264.7 cells were transfected with GSK3β-siRNA or negative control, and simultaneously cultured in osteoclast differentiation medium for 3 days. qRT-PCR analysis of the expression of *Gsk3β, Ctsk, Oscar, Mmp9* and *Nfatc1* (D) (n = 3); The protein level of GSK3β, ATP6V0D2, CTSK, NFATc1 and MMP9 was determined by western blot (E); GAPDH was used as an internal control. ^*^*p* < 0.05 for vs NC + DMSO or NC + pH7.4.

**
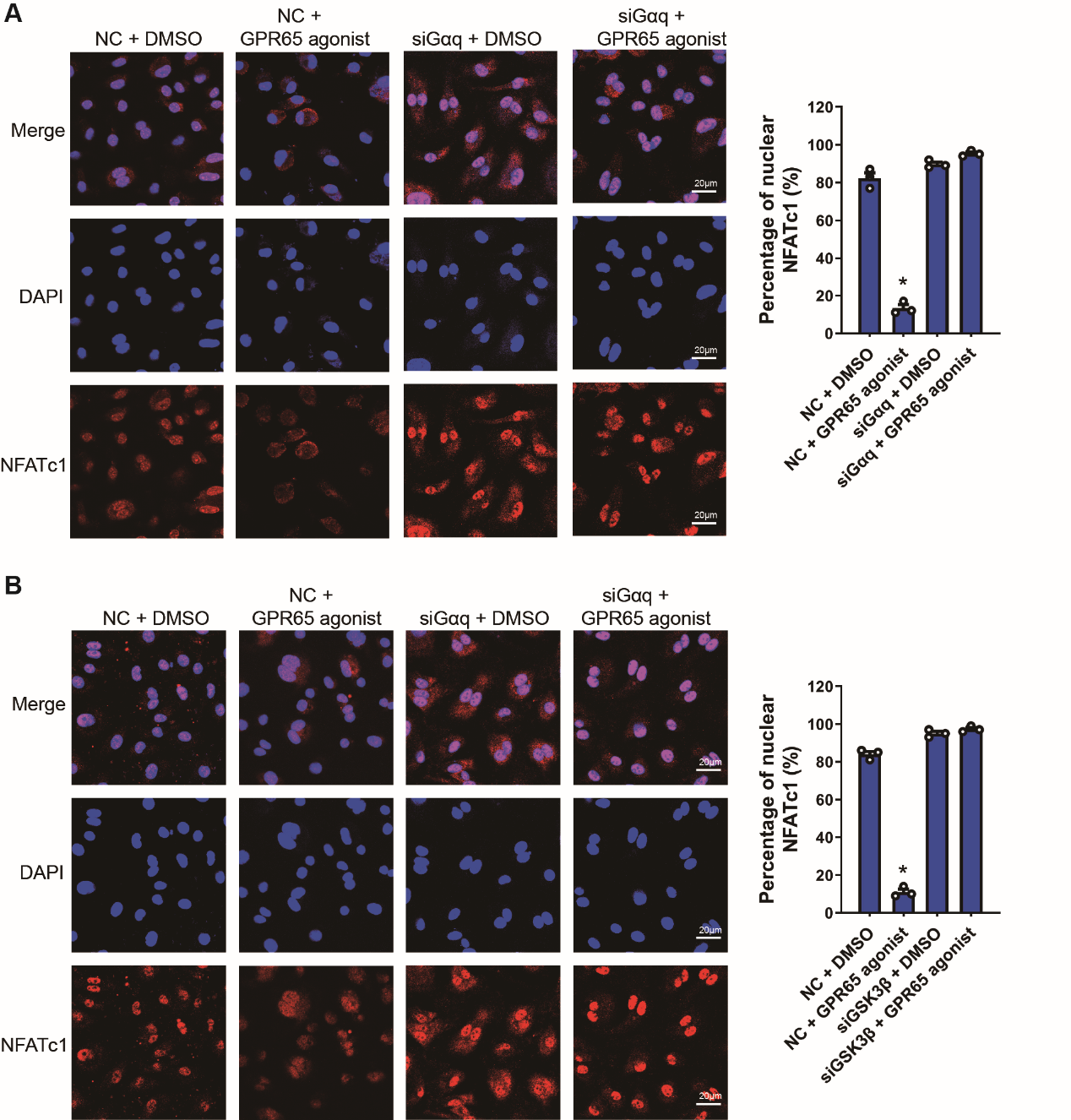
**

**Figure S15. GPR65 suppresses the expression and nuclear translocation of NFATc1 via Gαq and GSK3β, related to Figure 7**

(A) DMSO- or BTB09089- treated RAW264.7 cells were transfected with Gαq-siRNA or negative control, and simultaneously cultured in osteoclast differentiation medium for 3 days. The expression and location of NFATc1 was assessed by confocal microscopy; scale bar, 20 μm. Quantification of NFATc1 more nuclear (N > C) or more cytosolic (N < C) is determined with randomly chosen fields, each with approximately 100 cells. (B) DMSO- or BTB09089- treated RAW264.7 cells were transfected with GSK3β-siRNA or negative control, and simultaneously cultured in osteoclast differentiation medium. The expression and location of NFATc1 was assessed by confocal microscopy; scale bar, 20 μm. Quantification of NFATc1 more nuclear (N > C) or more cytosolic (N < C) is determined with randomly chosen fields, each with approximately 100 cells.

**
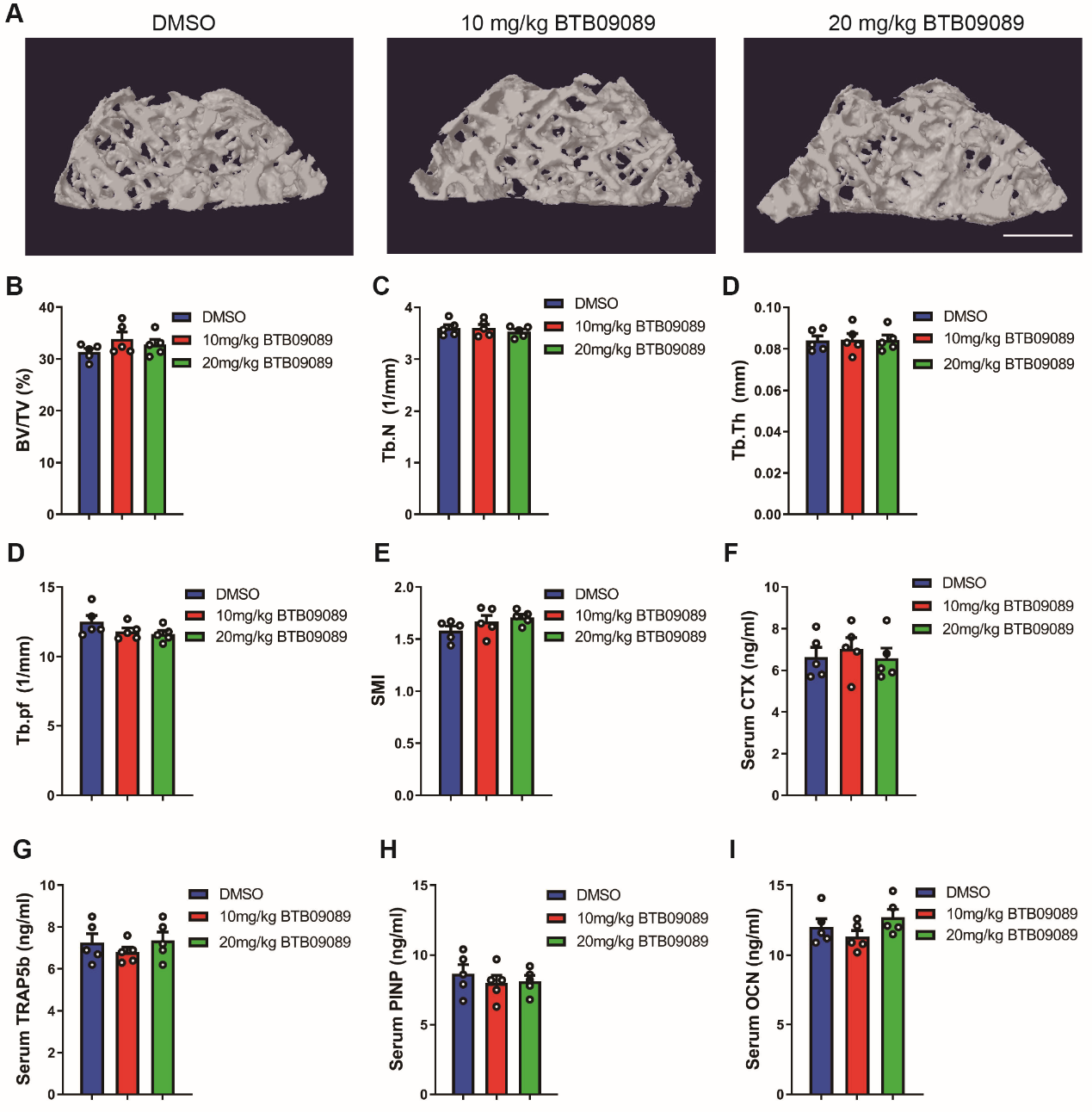
**

**Figure S16. Intraperitoneal injection of BTB09089 does not affect bone metabolism, related to Figure 8**

(A-I) Balb/c female mice were separated into 3 groups randomly (n = 5): DMSO, 10 mg/kg BTB09089 and 20 mg/kg BTB09089. BTB09089 was administered every two days for 2 months. Micro-CT analysis of distal femurs for BV/TV, Tb.N, Tb.Th, Tb. Pf and SMI (A-E). (F-I) Serum CTX, TRAP5b, PINP and OCN levels (A-E).

**
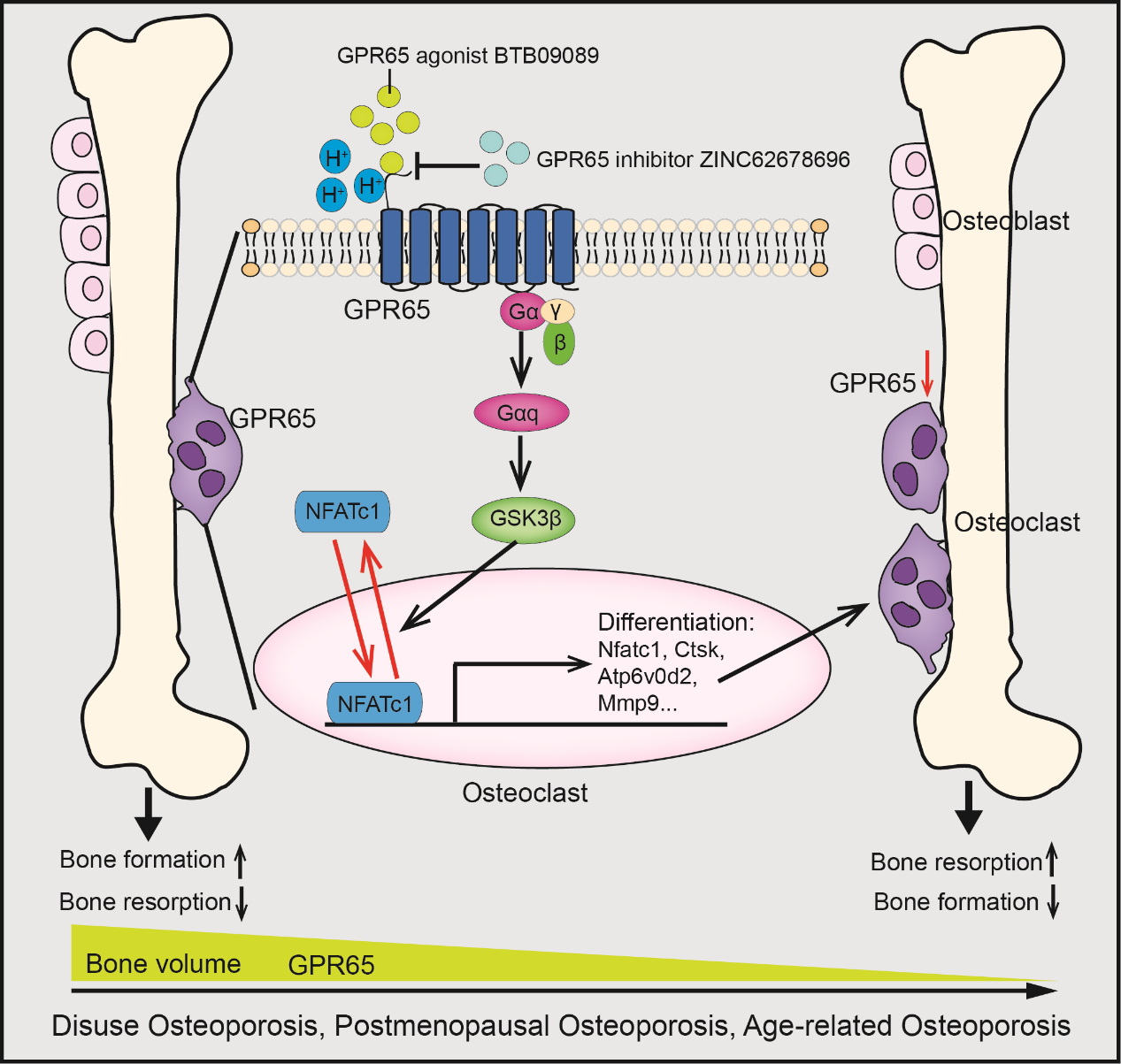
**

**Figure S17.** **Schematic diagram illustrating the function and mechanism of GPR65 in regulating osteoclast differentiation and osteoporosis.**

During osteoclast differentiation and the process of osteoporosis, the proton-sensing receptor GPR65, which is specifically expressed in osteoclasts within bone tissue and is significantly reduced. Its activation of the downstream Gαq-GSK3β pathway is inhibited, which in turn promotes the nuclear translocation and increased expression of NFATc1, facilitating osteoclast differentiation and the progression of osteoporosis. In contrast, the specific agonist for GPR65 can inhibit this process.

**Supplemental Tables and Table Legends**

**Table S1** siRNA sequences

| **Name** | **Forward (5’ – 3’)** | **Reverse (5’ – 3’)** |
| --- | --- | --- |
| *Gpr65-*siRNA-1 | GCGCCACCUCGAACACUAUTT | AUAGUGUUCGAGGUGGCGCTT |
| *Gpr65-*siRNA*-*2 | CCUGUCAGACCUGCUGUAUTT | AUACAGCAGGUCUGACAGGTT |
| *Gαq* | CCACAGAUACCGAGAACAUTT | AUGUUCUCGGUAUCUGUGGTT |
| *Gsk3β* | ACUCAAGAACΜGUCAAGUATT | UACUUGACAGUUCUUGAGUTT |
| Negative control | UUCUCCGAACGUGUCACGUTT | ACGUGACACGUUCGGAGAATT |

**Table S2** Quantitative real-time reverse transcription-polymerase chain reaction **(**qRT-PCR) primers for analysis of transcript levels

| **Gene symbol** | **Forward (5’ – 3’)** | **Reverse (5’ – 3’)** |
| --- | --- | --- |
| *mGapdh* | GGCATGGACTGTGGTCATGAG | TGCACCACCAACTGCTTAGC |
| *mGpr65* | CTGGACCGCTATTTAGCAGTC | GCTGGTAATAAACGCGAATCTTC |
| *mCtsk* | GAAGAAGACTCACCAGAAGCAG | TCCAGGTTATGGGCAGAGATT |
| *mOscar* | TGGCGGTTTGCACTCTTCA | GATCCGTTACCAGCAGTTCCAGA |
| *mMmp9* | CGTGTCTGGAGATTCGACTTGA | TTGGAAACTCACA CGCCAGA |
| *mNfatc1* | ACGCTACAGCTGTTCATTGG | CTTTGGTGTTGGACAGGATG |
| *mOcn* | GAGGGCAATAAGGTAGTGAA | CATAGATGCGTTTGTAGGC |
| *mGαq* | GGTCGGGCTACTCTGACGA | ACTTGTATGGGATCTTGAGCGT |
| *mGsk3β* | AGCTCTGATTGGCCACTGTC | CGTTACAGGCGTCTGGGAAT |
| *hGDPDH* | AATGGGCAGCCGTTAGGAAA | GCGCCCAATACGACCAAATC |
| *hGPR65* | ACAGCAATTCTGGGAAGCGA | ATTACACCTCCCAGTGCAGAA |
